# Customizable Open-Source Rotating Rod (Rotarod) Enables Robust Low-Cost Assessment of Motor Performance in Mice

**DOI:** 10.1523/ENEURO.0123-23.2023

**Published:** 2023-09-01

**Authors:** Josephine H. Widjaja, Douglas C. Sloan, Joseph A. Hauger, Brian S. Muntean

**Affiliations:** 1 Department of Pharmacology and Toxicology, Medical College of Georgia, Augusta University, Augusta, GA 30912; 2 Department of Chemistry and Physics, College of Science and Mathematics, Augusta University, Augusta, GA 30912

**Keywords:** coordination, motor learning, mouse behavior, open-source tool, rotarod

## Abstract

Reliable measurements of motor learning and coordination in mice are fundamental aspects of neuroscience research. Despite the advent of deep-learning approaches for motor assessment, performance testing on a rotating rod (rotarod) has remained a staple in the neuroscientist’s toolbox. Surprisingly, commercially available rotarod instruments offer limited experimental flexibility at a relatively high cost. In order to address these concerns, we engineered a highly-customizable, low-budget rotarod device with increased functionality. Here, we present a detailed guide to assemble this rotarod using simple materials. Our apparatus incorporates a variation of interchangeable rod sizes and designs which provides for adjustable testing sensitivity. Moreover, our rotarod is driven by open-source software enabling bespoke acceleration ramps and sequences. Finally, we report the strengths and weaknesses of each rod design following multiday testing on cohorts of C57BL/6 mice. We expect explorations in deviant rod types to provide a foundation for the development of increasingly sensitive models for motor performance testing along with low-budget alternatives for the research community.

## Significance Statement

We developed a low-cost and highly customizable rotarod for testing motor learning behavior in mice. We present a detailed assembly guide and open-source software for operation while demonstrating utility on cohorts of C57/B6 mice. We are hopeful this will be a promising step to innovate standard rotarod approaches while also significantly reducing economic barriers toward behavioral neuroscience.

## Introduction

Motor learning requires the acquisition of information pertaining to locomotor activity and the output to produce skilled motor functions. These processes require the integration of sensory signals with motor commands which incorporates multiple nuclei in the brain (Wolpert and Ghahramani, 2000; Arber and Costa, 2022). Skilled learning is thought to require synchronization between the motor cortex, striatum, and cerebellum (Penhune and Steele, 2012). Moreover, numerous genetic factors have been associated with motor learning impairments (Breakefield et al., 2008; Manto and Jissendi, 2012; Vicente et al., 2020). Rodent models have been fundamental in understanding the mechanics of motor learning (Sousa et al., 2006). A key resource in this regard is the behavioral testing of motor learning on an instrument that consists of an elevated accelerating rotating rod (or rotarod; Jones and Roberts, 1968).

The rotarod performance test developed by Dunham and Miya has indeed remained a hallmark for quantifying motor dysfunction in mice (Dunham and Miya, 1957). In this test, rodents are timed for their ability to maintain balance on the rotating rod. Repeated trials lead to increased latency to fall from the rod which provides a reliable indicator for motor learning. Thus, this behavioral assessment has profoundly influenced multiple facets of neuroscience research including neurodegeneration (Monville et al., 2006; Shiotsuki et al., 2010; Nampoothiri et al., 2017), pharmacology (Pearl et al., 1969; Cartmell et al., 1991; Ayton et al., 2013; Lubrich et al., 2022), and the genetic basis of disease (Chen et al., 1995; Costa et al., 2003; Oleas et al., 2013; Sutton et al., 2019; Muntean et al., 2021, 2022). Despite the extensive use of the rotarod, very few modifications have been made to the existing model as it has maintained its rigid structure and functions with only slight improvements to user controls and fall-time measuring systems. The standard commercial model of the rotarod generally consists of a 3-cm rotating horizontal rod supporting vertical horizontal barriers to simultaneously test multiple mice (Deacon, 2013). The implications of an unevolved rotarod may profoundly influence the ability to observe motor traits in different systems and inhibit the expansion of solutions for motor-related diseases. For instance, it has long been appreciated that rod diameter greatly influences rodent performance on the rotarod (Rozas et al., 1998; Rustay et al., 2003; Shiotsuki et al., 2010). Smaller diameter drums may encourage passive behavior as animals can cling to the rod during rotation (Rozas et al., 1998; Shiotsuki et al., 2010). On the other hand, larger diameter rods may also reflect walking/running ability in addition to traits of balance and coordination (Rustay et al., 2003). However, performing behavior experiments on different rod types is not common partly because of the inability to easily customize standard configurations of commercial systems. Moreover, commercially available instruments may also present an economic barrier to research progress.

Here, we describe a highly adjustable custom rotarod device for mice. We provide open-source code that can be modified in alignment with research goals as well as detailed instructions for the build that can be achieved at a low cost with simple components. Our design includes four interchangeable rod types and the ability to integrate an assortment of acceleration sequences, directions, and speeds. Our study defines the parameters and capabilities of this rotarod while exploring the influences of new modes of testing on mouse learning and motor ability. We hope to provide a cost-efficient variant for wider adoption of standard research instrumentation with enhanced customization to adapt with experimental demands in sensorimotor-related research.

## Materials and Methods

### Base assembly

**Figure 1.**
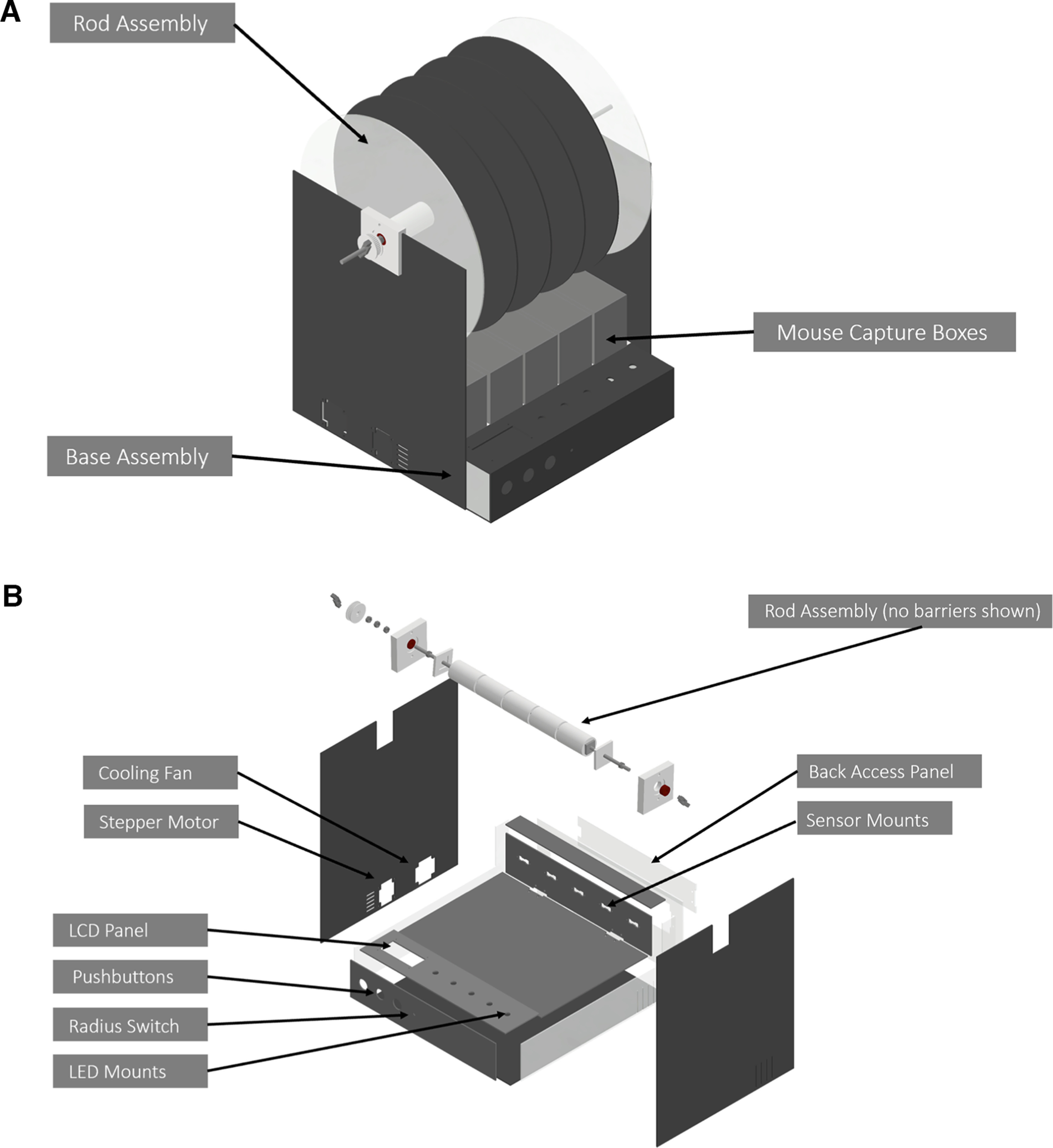
Schematic of base assembly of custom behavior device. ***A***, Unexpanded CAD modeled view of the base and rod assembly along with mouse capture boxes, electronic components not shown. ***B***, Expanded view of the 12 laser-cut parts of CAD modeled base, location of electronic components within the device are labeled. An expanded view of rod assembly components without the horizontal barriers is also shown. Assembly displays the 10 additional 3D-printed components (not including the rod), five nuts, and two winged nuts. Further details can be found in Extended Data Figures 1-1, 1-2, 1-3, 1-4, and 1-5.

The base, or main frame of the custom rotarod device, was synthesized from 1/8th-inch acrylic cut to form the individual base parts through laser-cutting technology (Fig. 1*A*). The base assembly is formed from 12 unique parts (not including the mouse capture boxes and tracks) that function to mount the sensors, liquid crystal display (LCD) panels, motor, LEDs, pushbuttons, fan, and switches (Fig. 1*B*). An overview of these parts, their relative dimensions, and CAD/laser cut files can be found on our GitHub repository (https://github.com/MunteanLab/mouseduino). The base contains a hinged lid and back panel that allows for easy access to relevant electrical components. These articulations are formed through an attachment of hinges, nuts, and bolts. A full description of materials can be seen in Table 1. Acrylic parts were bonded through superglue though we recommend using an acrylic bonding material for more resiliency and strength. To clean the acrylic panels, we recommend using bleach-based products such as Clorox. To keep waste products from falling into the device while the lid was opened, tape was used to cover spaces formed between the Lid Top Part A and Front Top part during the opening of the lid. These additional views and perspectives of the design are presented in Extended Data Figures 1-1 and 1-2. Assembly of the individual base parts can be seen in Extended Data Figures 1-3 and 1-4.

**Table 1:** Base assembly parts

Quantity	Item	Unit cost	Cost	Link
Rod assembly				
2	5/16th bolt (5 inches)	$0.69	$1.38	https://www.homedepot.com/p/Everbilt-5-16-in-18-x-5-in-Zinc-Plated-Hex-Bolt-800796/204645557
6	5/16th nuts	$0.46	$2.76	https://www.homedepot.com/p/Everbilt-5-16-in-18-Stainless-Steel-Hex-Nut-3-Pack-800061/204746982
2	5/16th winged nut	$0.99	$1.98	https://www.homedepot.com/p/Everbilt-5-16-in-18-Stainless-Wing-Nuts-15-Pack-38962/203436320
2	Ball bearing	$0.5	$1	https://www.amazon.com/%EF%BC%BB10-Pack%EF%BC%BD-608-Ball-Bearings/dp/B08XVFSZTF/ref=asc_df_B08XVFSZTF/?tag=hyprod-20&linkCode=df0&hvadid = 564681743706&hvpos=&hvnetw=g&hvrand = 12548061986415233110&hvpone=&hvptwo=&hvqmt=&hvdev=c&hvdvcmdl=&hvlocint=&hvlocphy = 9011161&hvtargid=pla-1334327078771&psc = 1&region_id = 373786
4	M5 nut	$0.625	$2.5	https://www.homedepot.com/p/M5-0-8-x-25-mm-Class-8-8-Zinc-Plated-Hex-Bolt-2-Pack-801378/204273538
4	M5 bolt	$0.625	$2.5	https://www.homedepot.com/p/5-mm-0-8-Stainless-Steel-Metric-Hex-Nut-2-per-Pack-801008/204274112
1	Black acrylic (18 × 18 inches)	$63.5	$63.5	https://www.homedepot.com/p/Falken-Design-36-in-x-36-in-x-1-8-in-Thick-Acrylic-Black-Opaque-Sheet-Falken-Design-ACRYLIC-BK-1-8-3636/308669330
1	Clear acrylic (36 × 36 inches)	$43.88	$43.88	https://www.homedepot.com/p/Falken-Design-36-in-x-36-in-x-1-8-in-Thick-Acrylic-Clear-Sheet-Falken-Design-ACRYLIC-CL-1-8-3636/308669890&corid=cd0257e4-9334-0e3d-4781-f777b9cd1fdf
Base assembly				
1	Black acrylic (24 × 72 inches)	$73.51	$73.51	https://www.homedepot.com/p/Falken-Design-24-in-x-72-in-x-1-8-in-Thick-Acrylic-Black-Opaque-Sheet-Falken-Design-ACRYLIC-BK-1-8-2472/308669309&corid=f630b29c-1ba0-c5cf-dc35-a7b99e10a33a
3	Loctite superglue	$4.27	$12.81	https://www.amazon.com/Loctite-Control-4-Gram-Bottle-1739050/dp/B00ELV2D0Y/ref=sxin_17?asc_contentid=amzn1.osa.d78b6cd0-30e6-4ba9-a22f-ca82e589a421.ATVPDKIKX0DER.en_US&asc_contenttype=article&ascsubtag=amzn1.osa.d78b6cd0-30e6-4ba9-a22f-ca82e589a421.ATVPDKIKX0DER.en_US&content-id=amzn1.sym.2501e731-e00e-46aa-97f8-28a8de3ef511%3Aamzn1.sym.2501e731-e00e-46aa-97f8-28a8de3ef511&creativeASIN=B00ELV2D0Y&cv_ct_cx=super+glue&cv_ct_id=amzn1.osa.d78b6cd0-30e6-4ba9-a22f-ca82e589a421.ATVPDKIKX0DER.en_US&cv_ct_pg=search&cv_ct_we=asin&cv_ct_wn=osp-single-source-pecos-desktop&keywords=super+glue&linkCode=oas&pd_rd_i=B00ELV2D0Y&pd_rd_r = 88ab3c4f-74e3-4322–9253-74cfdcbfb569&pd_rd_w=pK9wq&pd_rd_wg=EUobK&pf_rd_p = 2501e731-e00e-46aa-97f8-28a8de3ef511&pf_rd_r=NVQQ0SFJZHQ2NEJR1KM5&qid = 1675976921&sr = 1-4-c26ac7f6-b43f-4741-a772-17cad7536576&tag=bobvila-20
4	M2 bolts (LCD)	$0.025	$0.1	https://www.amazon.com/HanTof-Threaded-Machine-Assortment-Wrench%EF%BC%8CBlack/dp/B09D8ZZ9YM/ref=asc_df_B09D8ZZ9YM/?tag=hyprod-20&linkCode=df0&hvadid = 563795747675&hvpos=&hvnetw=g&hvrand = 9563969514790772077&hvpone=&hvptwo=&hvqmt=&hvdev=c&hvdvcmdl=&hvlocint=&hvlocphy = 9011161&hvtargid=pla-1634653412313&th = 1
4	M2 nuts	$0.025	$0.1	https://www.amazon.com/HanTof-Threaded-Machine-Assortment-Wrench%EF%BC%8CBlack/dp/B09D8ZZ9YM/ref=asc_df_B09D8ZZ9YM/?tag=hyprod-20&linkCode=df0&hvadid = 563795747675&hvpos=&hvnetw=g&hvrand = 9563969514790772077&hvpone=&hvptwo=&hvqmt=&hvdev=c&hvdvcmdl=&hvlocint=&hvlocphy = 9011161&hvtargid=pla-1634653412313&th = 1
4	M5 bolts 40 mm (stepper motor)	$1.375	$5.5	https://www.homedepot.com/p/Everbilt-M5-0-8-x-40-mm-Phillips-Pan-Head-Stainless-Steel-Machine-Screw-2-Pack-843098/204283788
8	M5 nuts	$0.625	$5	https://www.homedepot.com/p/5-mm-0-8-Stainless-Steel-Metric-Hex-Nut-2-per-Pack-801008/204274112
4	M5 bolt 50 mm (fan)	$1.375	$5.5	https://www.homedepot.com/p/Everbilt-M5-0-8-x-50-mm-Phillips-Pan-Head-Stainless-Steel-Machine-Screw-2-Pack-843118/204283790
12	(Hinges) #6–32 × 3/14-inch flat Phillips and nNuts	$0.267	$3.204	https://www.homedepot.com/p/Hillman-6-32-x-3-4-in-Phillips-Flat-Head-Machine-Screws-25-Pack-4058/204795038
1	Acrylic clasps	$8.99	$8.99	https://www.amazon.com/gp/product/B0814QLSJF/ref=ppx_yo_dt_b_asin_title_o01_s00?ie=UTF8&psc=1
20	Miniwheels	$0.458	$9.16	https://www.amazon.com/gp/product/B08ZHGW6TX/ref=ppx_yo_dt_b_asin_title_o01_s00?ie=UTF8&psc=1

Total cost $247.37.

10.1523/ENEURO.0123-23.2023.f1-1 Extended Data Figure 1-1 Base design assembly. Schematic overview of the base with views from the right, left, and back. Download Figure 1-1, TIF file.

**Figure 2.**
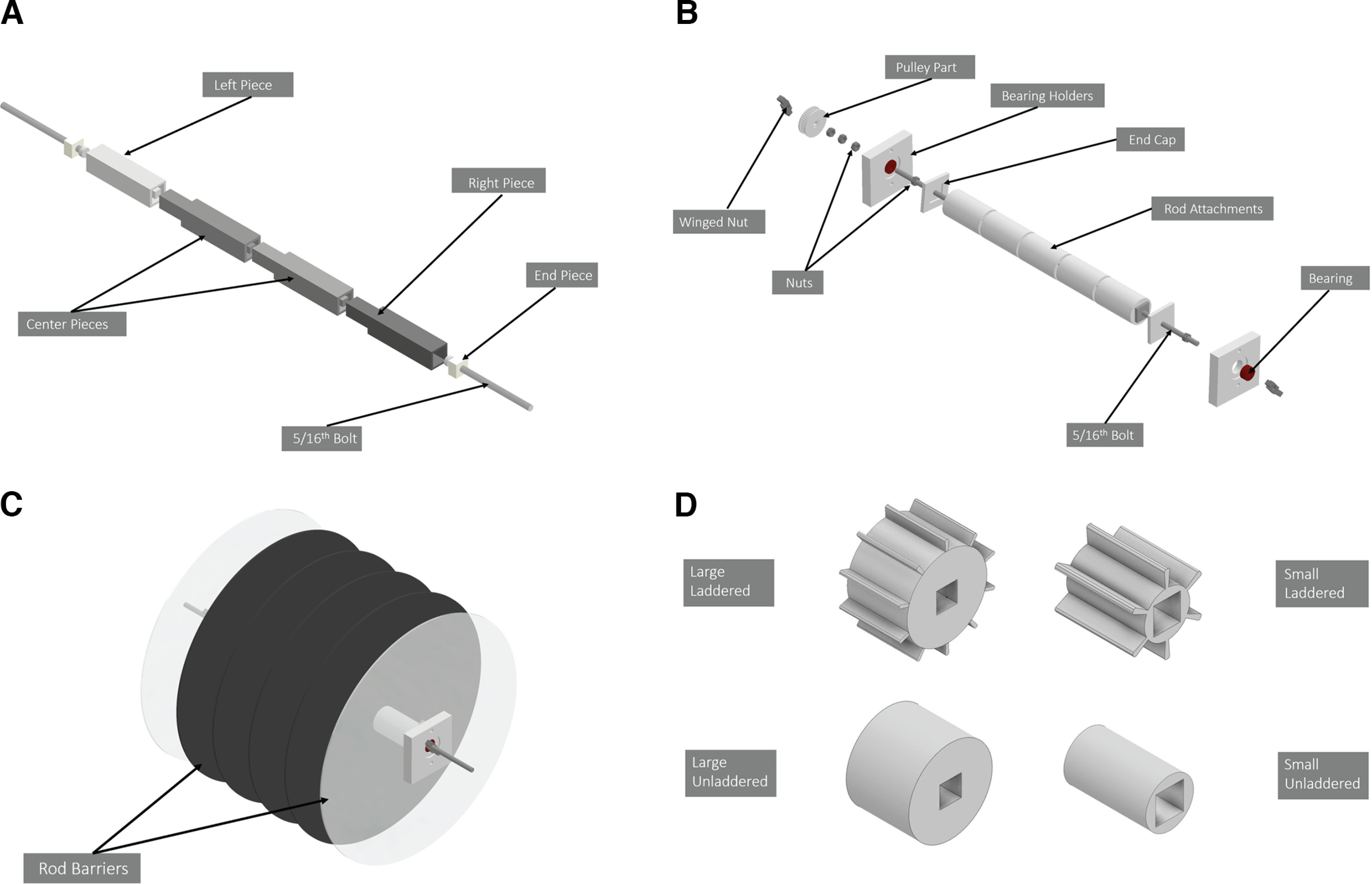
Breakdown of rod assembly including the rod and rod attachments. ***A***, Expanded CAD modeled view displaying components for the rod consisting of six 3D-printed components secured through superglue and two 5/16th bolts. ***B***, Components of the rod attachments. ***C***, Unexpanded view of complete rod assembly. ***D***, Various drums sizes and diameters made for use with our interchangeable rod model. Further details can be found in Extended Data Figures 2-1 and 2-2.

10.1523/ENEURO.0123-23.2023.f1-2 Extended Data Figure 1-2 Expanded schematic views of base rotarod. Download Figure 1-2, TIF file.

**Figure 3.**
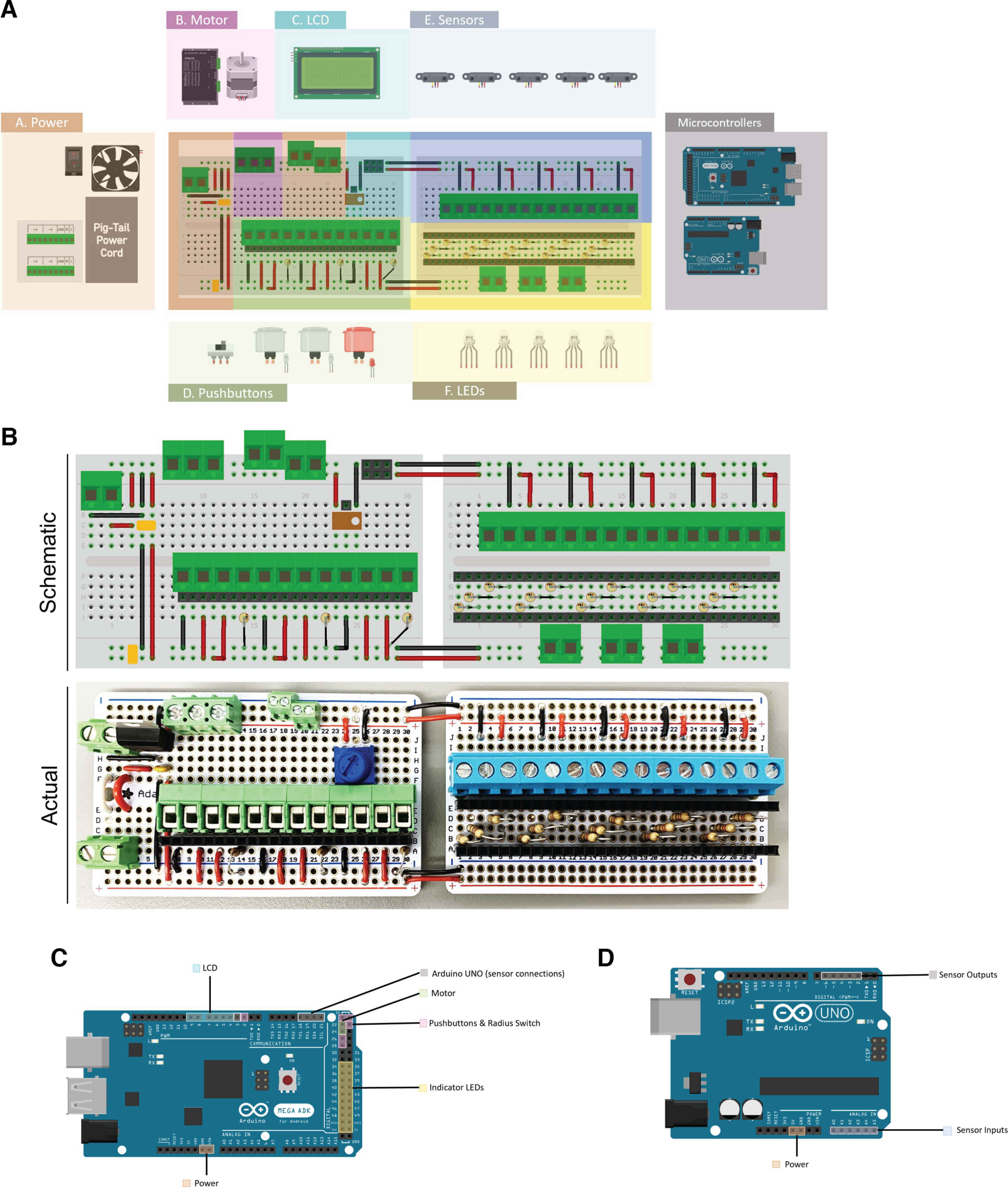
Overview of key electronic components. ***A***, Color-coded breadboard schematic with associated electrical components. Each color represents the general region of attachment of the component to the breadboard. ***B***, Wiring diagram of breadboard with schematic (top) and example of an assembled board (bottom). ***C***, Arduino MEGA, main-microcontroller, pin-attachment schematic. ***D***, Arduino UNO, secondary microcontroller, pin-attachment schematic.

10.1523/ENEURO.0123-23.2023.f1-3 Extended Data Figure 1-3 Labeled components on the base. Download Figure 1-3, TIF file.

**Figure 4.**
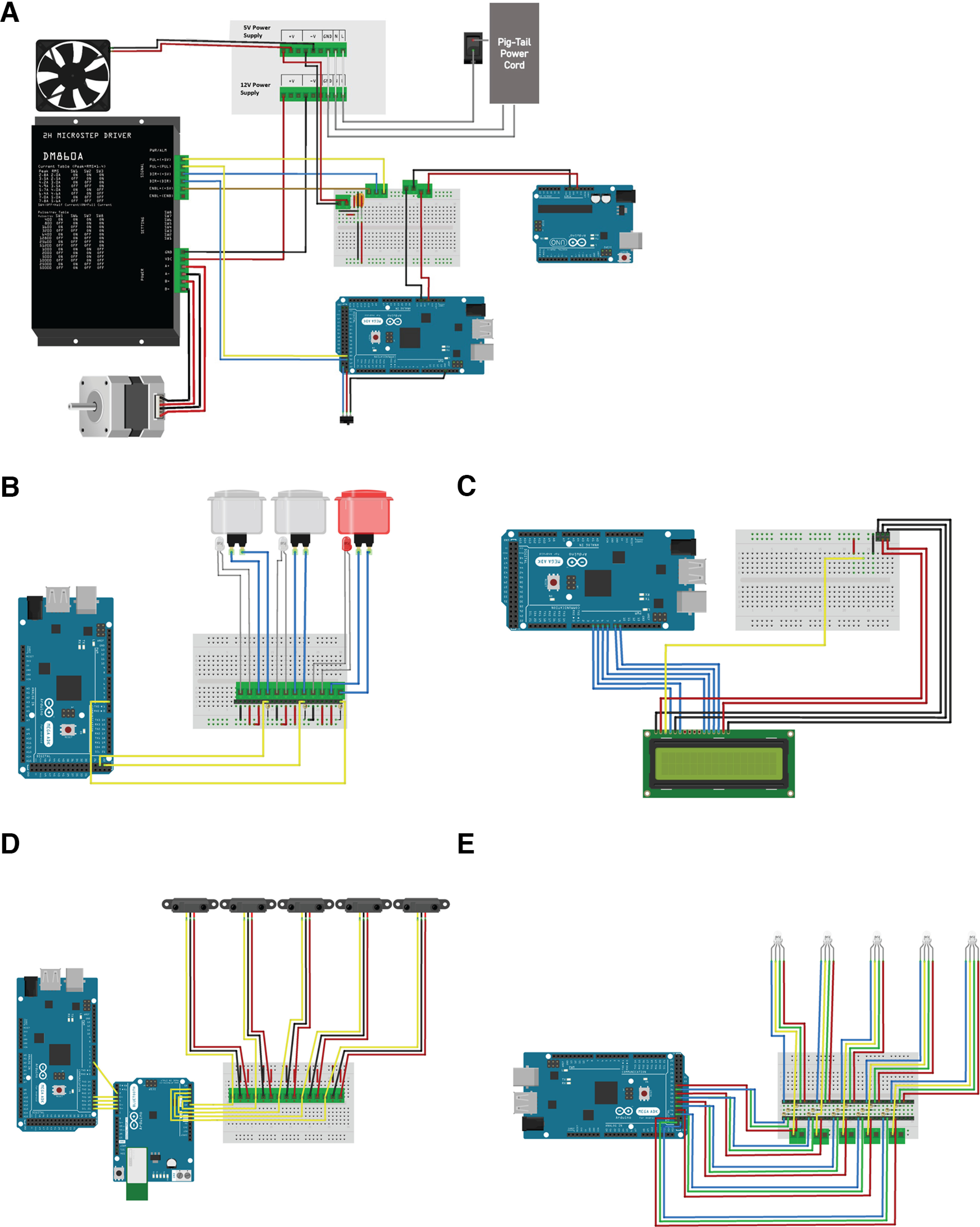
Wiring diagrams of electrical components using Fritzing editor. Each diagram represents the wiring schematic based on the breadboard in Figure 3. Only breadboard components directly attached to each part are shown in the image. Wire length and position are not to scale. Each diagram presents the relevant attachments to the two microcontrollers. ***A***, Wiring of power supply and related switches, stepper motor attachments, fan, radius switch, and relevant connections to associated microcontrollers. ***B***, Wiring diagram of pushbuttons and relevant connections to associated microcontrollers. LEDs in this diagram are representative of the light-related connectors within the pushbutton not shown in the pushbutton image. ***C***, Wiring diagram of LCD panel and associated microcontroller connections. ***D***, Wiring diagram of sensors and associated microcontroller connections. ***E***, Wiring diagram of RGB LEDs and associate microcontroller connections. Further details can be found in Extended Data Figure 4-1.

10.1523/ENEURO.0123-23.2023.f1-4 Extended Data Figure 1-4 Rod barriers. The barriers on the rod are shown in part L. The design requires six rods with recommendation for two clear and four opaque barriers. Acrylic dimensioning is also shown. Acrylic sheets were cut down to fit inside our laser cutter. Download Figure 1-4, TIF file.

**Figure 5.**
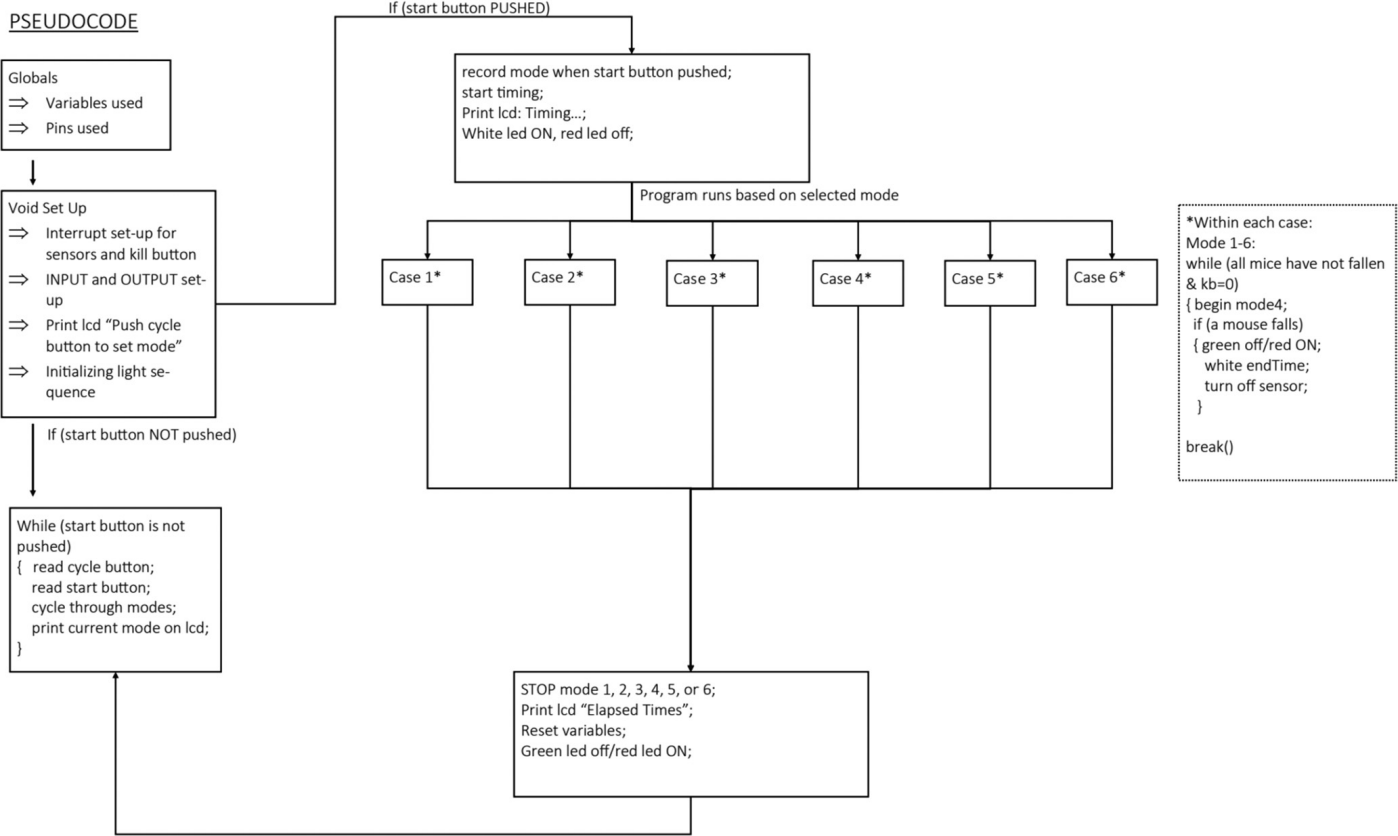
Pseudocode structure for code. This figure demonstrates flow and logic of code written and integrated with Arduino IDE.

10.1523/ENEURO.0123-23.2023.f1-5 Extended Data Figure 1-5 Mouse capture box design. ***A***, Mouse capture boxes shown with dimensions and were cut from 1/8-inch acrylic sheets. A total of five are required. ***B***, Final view of an assembled capture box. ***C***, Mouse capture box tracks shown with dimensions and were cut from 1/8-inch acrylic sheets. A total of five is required. ***D***, Final view of tracks for the capture boxes. Download Figure 1-5, TIF file.

10.1523/ENEURO.0123-23.2023.f2-1 Extended Data Figure 2-1 Rod attachment designs. Designs for the four unique rod models developed. Note that the large-laddered rod was printed in one large piece and ten notched pieces. The notches piece dimensions were synthesized by cutting the notched pieces from a nonseparated laddered rod drawing. Download Figure 2-1, TIF file.

10.1523/ENEURO.0123-23.2023.f2-2 Extended Data Figure 2-2 Actual images of rod assembly. Download Figure 2-2, TIF file.

10.1523/ENEURO.0123-23.2023.f4-1 Extended Data Figure 4-1 Actual images of rotarod assembly. ***A***, Images of base assembly. ***B***, Images of electronics assembly. Download Figure 4-1, TIF file.

### Mouse capture boxes

We developed open-top boxes from 1/8th-inch acrylic composed of four laser cut pieces. To these boxes, four plastic wheels were superglued onto the acrylic. Each pair of the plastic wheels, placed both on the front and back ends of the capture box, were separated by a little over an inch to accommodate for a one-inch acrylic track that was attached to the hinging lid (Lid Top Part A) of the base assembly. Dimensions for the position of the tracks on the lid along with additional capture box assembly instruction can be found in Extended Data Figure 1-5 as well as on our GitHub repository. The bottom of the capture box was lined with a piece of foamboard wrapped with packaging tape. An additional removable layer of paper was added to catch waste throughout the experiment. This contaminated layer was removed after each use.

### Rod assembly

Using 3D-printing and laser-cutting tools, we synthesized a custom rod that holds detachable and interchangeable rod drum sizes and shapes (Fig. 2*A*). Our 3D-printed parts were made from standard polylactic acid 3D-printer filament. 3D-printed files were created using AutoDesk Inventor, a computer design application for 3D modeling and visualization, and were printed by a local printing facility. Our rod design has a cuboid shaft designed for homologous rotation of the rod attachments. It consists of four 3D-printed parts that lock through male and female ends. To each end of the rod, a cuboid end piece with a hole accommodating a five-inch 5/16th bolt was superglued. The bolts were oriented such that the body of the nut faced the external side of the rod and the head of the nut was embedded in the interior. Five of the various 3D-printed rod attachments (small or large/plain or laddered) and six laser-cut circular chamber dividers were placed onto the rod in an alternating fashion (divider, rod attachment, divider, rod attachment…) with clear dividers located at the ends to accommodate future modifications (Fig. 2*B*,*C*). These parts were secured with a 3D-printed end cap. The rotating capability of the rod was synthesized by threading the five-inch bolt into a ball bearing nested and superglued into a 3D-printed component (termed “bearing holders”). These bearing holders consist of two separate units joined together by two M5 nuts and their corresponding bolts. Aside from enabling rotation, these holders also attach and suspend the rod assembly to the main frame of the device.

The static end of the rod (the left end as seen on Fig. 2*B*), contains a pulley part (labeled as “top pulley” in our GitHub repository) with embedded bolts that connect the rod assembly to the motor that also holds a 3D-printed pulley attachment on its hub (“bottom pulley”). The pulley part consists of a 5/16th nut embedded into a 3D-printed part, thus allowing it to be screwed onto the 5/16th bolt protruding from the main body of the rod. The dynamic end (the right end as seen on Fig. 2*B*), was used to access and interchange the rod attachments through unscrewing attached nuts and sliding rod components off the shaft. To the static end, these items were attached following the end cap (listed from most interior to exterior position): a 5/16th bolt, the bearing assembly, three 5/16th bolts, the pulley part, and one winged nut. To the dynamic end, the following was attached following the end cap: one 5/16th nut, the bearing part, and one winged nut. A complete list of the materials used to synthesize the rod assembly can be observed in Table 1.

A core innovation in our rotarod is the ability to interchange various rod designs. Here, we describe two different drum sizes for two unique models (laddered and unladdered; Fig. 2*D*; Extended Data Fig. 2-1, 2-2). The small unladdered design contains a 4.445 cm in diameter drum, which reflects a typical size range used in many commercial applications. We also included a larger diameter drum (10.16 cm) which we hypothesized may provide additional challenge and thus sensitivity to motor learning. Finally, we describe a laddered version of both rods (small and large) that requires animals to specifically grab onto the rung rather than at a random placement. 3D printing, laser cutting files, images, and supplementals with more information on the rod assembly can be accessed on our GitHub repository mentioned above. Our current assembly can accommodate rod drums of 1.5 inches and greater. However, this can be adapted by creating a smaller rod shaft through 3D-printing technology if smaller drum sizes are desired.

### Electronics assembly

#### Overview

The electronics assembly integrates user input and measurement output while including seven main components as shown in Figure 3*A* and described separately in the sections below. The main components were connected using several through-hole solder boards, 24-gauge solid and stranded wires, terminal blocks and custom-made DuPont connecting wires. See Figure 3*B* for breadboard connection diagrams and Figure 3*C* for a brief overview of the microcontroller pin connections. Specific wiring diagrams can be seen in Figure 4. These figures show the additional wiring connections relative to the breadboard connection diagram in Figure 3*B*. Images of the complete build are shown in Extended Data Figure 4-1. A list of parts and materials can be found in Table 2.

**Table 2:** Electronics assembly materials list

Quantity	Item	Unit cost	Total Cost	Link
LEDs				
5	Diffused RGB LED (common anode/10 mm/tri-color)	$1.00	$4.98	https://www.adafruit.com/product848
5	LED holder (10 mm)	$0.30	$1.50	https://www.adafruit.com/product/2171
Stepper motor				
1	NEMA 23 stepper motor (DM860A)	$27.99	$27.99	https://openbuildspartstore.com/nema-23-stepper-motor/?sku=518&gclid=CjwKCAiAoL6eBhA3EiwAXDom5uQpFuZldM2HkEw8qTKptm9qH-XDbMsBYn1wlJ_BNWEcP-beQz9JLxoCKTQQAvD_BwE
1	Stepper motor driver	$9.99	$9.99	https://www.amazon.com/OUYZGIA-Stepper-Segments-Upgraded-Version-1PCS/dp/B08PKJG2ND/ref=sr_1_1_sspa?gclid=Cj0KCQiA_bieBhDSARIsADU4zLdwD1w8hyHwTbSpKeD2rVw_IB2w8pOjKy2bUHXnMjyEFolxc4onTuQaAgStEALw_wcB&hvadid = 616990817417&hvdev=c&hvlocphy = 9011165&hvnetw=g&hvqmt=e&hvrand = 13381348442473219371&hvtargid=kwd-30514926&hydadcr = 24630_13611748&keywords=stepper%2Bmotor%2Bcontroller&qid = 1674528349&sr = 8–1-spons&spLa=ZW5jcnlwdGVkUXVhbGlmaWVyPUFPV0gySThGMDNHSVYmZW5jcnlwdGVkSWQ9QTA0NTc4MjczS1ZGV0tGVzdDRTlIJmVuY3J5cHRlZEFkSWQ9QTA0MDQ3MDkzTUhWUkkwUDhDMktIJndpZGdldE5hbWU9c3BfYXRmJmFjdGlvbj1jbGlja1JlZGlyZWN0JmRvTm90TG9nQ2xpY2s9dHJ1ZQ&th = 1
Sensors				
5	Sharp IR range sensor (GP2Y0A02YK0F/20–150 cm)	$10.45	$52.25	https://www.amazon.com/ELEGOO-ATmega2560-ATMEGA16U2-Projects-Compliant/dp/B01H4ZLZLQ/ref=sr_1_2_sspa?gclid=Cj0KCQiA_bieBhDSARIsADU4zLcS6eXNGWAINGfcoDVgKMnC42_354Z0Xqn9y6T-XLoOnvL-Arnsr9YaAiCtEALw_wcB&hvadid = 570507306139&hvdev=c&hvlocphy = 9011165&hvnetw=g&hvqmt=e&hvrand = 10337382385117367914&hvtargid=kwd-270409539501&hydadcr = 18006_13462244&keywords=elegoo%2Bmega%2B2560&qid = 1674528182&sr = 8–2-spons&spLa=ZW5jcnlwdGVkUXVhbGlmaWVyPUEzRzRWVDNFSFVVUjJaJmVuY3J5cHRlZElkPUExMDIzMTAxMTZNR0hYUkxPUUw4MiZlbmNyeXB0ZWRBZElkPUEwNDA1MjE3Mk5WUUhXTUtGMDVJOCZ3aWRnZXROYW1lPXNwX2F0ZiZhY3Rpb249Y2xpY2tSZWRpcmVjdCZkb05vdExvZ0NsaWNrPXRydWU&th = 1
Microcontrollers				
1	Arduino mega 2560 (ELEGOO compatible)	$20.99	$20.99	https://www.amazon.com/ELEGOO-ATmega2560-ATMEGA16U2-Projects-Compliant/dp/B01H4ZLZLQ/ref=sr_1_1_sspa?crid=M1PAK4CCUJC4&keywords=arduino%2Bmega&qid = 1674592889&sprefix=arduino%2Bmega%2Caps%2C128&sr = 8-1-spons&spLa=ZW5jcnlwdGVkUXVhbGlmaWVyPUFTR1dZNENXQ1dHN0cmZW5jcnlwdGVkSWQ9QTA2NTQxMjUxR001V0pHNU1HT0REJmVuY3J5cHRlZEFkSWQ9QTA0MDUyMTcyTlZRSFdNS0YwNUk4JndpZGdldE5hbWU9c3BfYXRmJmFjdGlvbj1jbGlja1JlZGlyZWN0JmRvTm90TG9nQ2xpY2s9dHJ1ZQ&th = 1
1	Arduino UNO (ELEGOO compatible)	$16.99	$16.99	https://www.amazon.com/ELEGOO-Board-ATmega328P-ATMEGA16U2-Compliant/dp/B01EWOE0UU/ref=sr_1_1_sspa?crid = 2CJAVA80GVL12&keywords=arduino+uno&qid = 1674528229&sprefix=arduino+uno%2Caps%2C127&sr = 8-1-spons&psc = 1&spLa=ZW5jcnlwdGVkUXVhbGlmaWVyPUEyN1BEWjdLMVpLTTdEJmVuY3J5cHRlZElkPUEwMzgyMjMzMkk0WDA5TkFCUjRLRCZlbmNyeXB0ZWRBZElkPUEwNDgzODMzMUlIN1I4WVRSM0w1UiZ3aWRnZXROYW1lPXNwX2F0ZiZhY3Rpb249Y2xpY2tSZWRpcmVjdCZkb05vdExvZ0NsaWNrPXRydWU=
Buttons and switches				
1	Large toggle switch (20 amps)	$4.76	$4.76	https://www.homedepot.com/p/Gardner-Bender-20-Amp-Single-Pole-Toggle-Switch-1-Pack-GSW-121/100117400?source=shoppingads&locale=en-US&&mtc=SHOPPING-CM-CML-GGL-D27-027_002_WIRING_DEVIC-NA-NA-NA-SMART-2997116-NA-NA-NA-NBR-NA-NA-NEW-PL3&cm_mmc=SHOPPING-CM-CML-GGL-D27-027_002_WIRING_DEVIC-NA-NA-NA-SMART-2997116-NA-NA-NA-NBR-NA-NA-NEW-PL3-71700000093390730–58700007789602690-92700070740570989&gclid=Cj0KCQiAic6eBhCoARIsANlox87aIf6_QyrNLHHahJ4B2Al4LPxqtHrTT5lfhgW3zwycTaaq0b_LwBQaApsdEALw_wcB&gclsrc=aw.ds
1	Small toggle switch (5 amps)	$1.59	$1.59	https://www.jameco.com/z/T100T1B1A1QN-Jameco-ValuePro-Mini-Toggle-Switch-SPST-ON-OFF-5A-125VAC_76523.html
2	White LED pushbutton (24 mm)	$2.50	$5.00	https://www.adafruit.com/product/3429
1	Red LED pushbutton (24 mm)	$2.50	$2.50	https://www.adafruit.com/product/3430
LCD panel				
1	20 × 4 standard LCD panel	$17.95	$17.95	https://www.adafruit.com/product/198
Power				
1	12-V power supply	$12.20	$12.20	https://www.jameco.com/z/LRS-35-12-MEAN-WELL-36W-12V-3A-Single-Output-Enclosed-Power-Supply_2257475.html
1	5-V power supply	$9.12	$9.12	https://www.jameco.com/z/RS-15-5-MEAN-WELL-AC-to-DC-Power-Supply-5-Volt-3-Amp-15-Watt_1919043.html
1	Wall plug	$6.42	$6.42	https://www.digikey.com/en/products/detail/qualtek/411006–01/13687867?utm_adgroup=Power%2C%20Line%20Cables%20and%20Extension%20Cords&utm_source=google&utm_medium=cpc&utm_campaign=Shopping_Product_Cable%20Assemblies_NEW&utm_term=&utm_content=Power%2C%20Line%20Cables%20and%20Extension%20Cords&gclid=CjwKCAiAoL6eBhA3EiwAXDom5lpABe7hwMlbIpKqKMEA38DmicBzHqGUSEJ7gYcF-tcDGDSbIbCUyRoC9PcQAvD_BwE
1	Spade connectors (kit)	$0.03	$0.03	https://www.amazon.com/Nilight-Disconnect-Electrical-Solderless-Connectors/dp/B07V35375X/ref=sr_1_4?crid=1L575SKF7EJHK&keywords=spade%2Bconnector%2Bkit&qid=1674593423&sprefix=spade%2Bconnector%2Bkit%2Caps%2C110&sr=8-4&th=1
Bread board				
2	Half-sized breadboard PCB	$4.50	$9.00	https://www.adafruit.com/product/1609?gclid=Cj0KCQiA_bieBhDSARIsADU4zLcjrpxeF4P4isKgfLrtBxQLvsXbniKkpfEIAtMdjugmabYGUZJLKLsaAiQmEALw_wcB
2	0.1-mF tantalum capacitors	$0.65	$1.30	https://www.digikey.com/en/products/detail/kyocera-avx/TAP104K050SCS/563935?utm_adgroup=Capacitors&utm_source=google&utm_medium=cpc&utm_campaign=Shopping_Supplier_AVX%20Corporation&utm_term=&utm_content=Capacitors&gclid=CjwKCAiAoL6eBhA3EiwAXDom5tu0SB96PVJ688eumegcFZsAci7DpuReyrp1EaUldpeKGV32YpPuRhoCJkUQAvD_BwE
1	Linear potentiometer 10K	$2.43	$2.43	https://www.digikey.com/en/products/detail/bourns-inc/3296W-1-103LF/1088045
3	1-KΩ through hole resistor (1/4 Watts)	$0.10	$0.30	https://www.digikey.com/en/products/detail/stackpole-electronics-inc/CF14JT1K00/1741314
15	220-Ω through hole resistor (1/4 Watts, carbon film)	$0.10	$1.50	https://www.digikey.com/en/products/detail/yageo/CFR-25JB-52-220R/1295?utm_adgroup=Through%20Hole%20Resistors&utm_source=google&utm_medium=cpc&utm_campaign=Shopping_Product_Resistors_NEW&utm_term=&utm_content=Through%20Hole%20Resistors&gclid=CjwKCAiAoL6eBhA3EiwAXDom5m71uzrUJFahuUs3fm_a_w7JEXvnr3Dyv-B3BQxjIqlFLaVK701ojhoC_fUQAvD_BwE
Wiring				
1	24-gauge stranded wires (kit)	$11.95	$11.95	https://www.amazon.com/Fermerry-Stranded-Electrical-Silicone-Cables/dp/B089CRSLG8/ref=asc_df_B089CRSLG8/?tag=hyprod-20&linkCode=df0&hvadid = 459709171878&hvpos=&hvnetw=g&hvrand = 8180558533934889136&hvpone=&hvptwo=&hvqmt=&hvdev=c&hvdvcmdl=&hvlocint=&hvlocphy = 9011161&hvtargid=pla-945107864775&th = 1
1	24-gauge solid core wire (kit)	$15.95	$15.95	https://www.digikey.com/en/products/detail/adafruit-industries-llc/1311/6198255?utm_adgroup=Cables%2C%20Wires%20-%20Single%20Conductors&utm_source=google&utm_medium=cpc&utm_campaign=Shopping_Product_Kits&utm_term=&utm_content=Cables%2C%20Wires%20-%20Single%20Conductors&gclid=CjwKCAiAoL6eBhA3EiwAXDom5re0YwCgnwsDSlFU5wqJSisD5GJU32PST4S4tHmEgv7IVBVRicjpHhoC50IQAvD_BwE
1	Custom made dupont cable (kit)	$9.99	$9.99	https://www.amazon.com/Dupont-Connector-Kit-Connectors-Plusivo/dp/B078RRPRQZ/ref=asc_df_B078RRPRQZ/?tag=hyprod-20&linkCode=df0&hvadid = 241994846339&hvpos=&hvnetw=g&hvrand = 6717711556440729557&hvpone=&hvptwo=&hvqmt=&hvdev=c&hvdvcmdl=&hvlocint=&hvlocphy = 9011161&hvtargid=pla-570006049811&psc = 1
1	Female/male header row (pack of 5)	$2.95	$2.95	https://www.adafruit.com/product/598
9	3 input terminal blocks (5.08 mm)	$1.22	$10.98	https://www.digikey.com/en/products/detail/on-shore-technology-inc/EDZ500-3DS/250583
3	2 input vertical terminal block (5.08 mm)	$1.98	$5.94	https://www.digikey.com/en/products/detail/phoenix-contact/1868128/2511083
2	2 input side terminal blocks (2.54 mm)	$1.95	$3.90	https://www.digikey.com/en/products/detail/te-connectivity-amp-connectors/282834-2/1150135
Other				
1	12-V DC Sunon Maglev cooling fan (ME60151V1-000U-A99)	$9.06	$9.06	eBay purchase
1	Mini zip ties (pack)	$3.85	$3.85	https://www.amazon.com/Bates-Zip-Ties-4-inch/dp/B08P2HKSVM/ref=asc_df_B08P2HKSVM/?tag=hyprod-20&linkCode=df0&hvadid = 533377409716&hvpos=&hvnetw=g&hvrand = 5167591836732584198&hvpone=&hvptwo=&hvqmt=&hvdev=c&hvdvcmdl=&hvlocint=&hvlocphy = 9011161&hvtargid=pla-1393112843963&psc = 1

Total cost $286.36.

#### Component power

A 5-V DC, 3A Meanwell RS-15-5 is used to power the Arduino Uno and Arduino MEGA boards (Fig. 4*A*). The power supply is connected to the ground and V_in_ connections on each microcontroller board. This power supply also drives the 5-V cooling fan (optional) we have installed in the device enclosure. The DM860A stepper motor controller receives power from a separate 12-V DC, 8.5-A Meanwell RS-100-12. This power is delivered directly to the stepper motor coils via terminal block connections on the motor controller housing. The power supplies are connected in parallel to line voltage through an ordinary three-prong power cord. The power to the entire system is controlled by a 20-A toggle switch.

#### Stepper motor

A NEMA-23 bipolar stepper motor is used to drive the rotarod apparatus (Fig. 4*A*). A nonslipping belt connects the stepper motor timing pulley to the 3D-printed pulley mounted on the rotarod axle. The stepper’s four leads are connected directly to the output terminal blocks of the stepper motor controller which causes the stepper to rotate or “step” in response to pulses received from the Arduino MEGA. The rotation rate is determined by the rate of these pulses. Additional details are included in the section describing Arduino MEGA programming. The stepper motor controller is powered by the 12-V DC power supply mentioned above.

#### User input pushbuttons

Three one-inch diameter lighted pushbuttons provide user input to the rotarod system. These three buttons are used to select one of six testing modes, to start a test, to override the program or to stop a test. Repeated pressing on the mode selection button cycles the user through the six testing modes. When the user sees the desired mode displayed on the LCD, pressing the start button begins the test in the selected mode. The test continues until the total time expires, all mice have fallen, or the stop button is pushed. At the completion of the test, the elapsed time or latency for each mouse is displayed on the LCD. The mode selection and start buttons are illuminated with internal white LEDs. The stop button is illuminated with an internal red LED. Each button is appropriately labeled on the enclosure. Wiring schematics for the user input pushbuttons can be seen in Figure 4*B*.

#### Sensors, indicators, and liquid crystal display

Our device uses five infrared range sensors to simultaneously test up to five mice. Each range sensor is interfaced to an on-board 10-bit analog to digital converter on the Arduino UNO microcontroller board. These sensors, which are mounted at the end of each testing lane, produce a voltage that varies approximately inversely with distance. Thus, a fallen mouse will cause the corresponding sensor’s output voltage to surge. These voltages are continuously polled by the Arduino UNO and if such a voltage increase is detected, a digital output line on the Arduino UNO is taken high. This digital output is connected to an interrupt input pin on the Arduino MEGA. When the interrupt signal is received, the Arduino MEGA stops the timing on that particular mouse and stores that time for display at the end of the testing. In addition, the Arduino MEGA changes the RGB LED indicator for that testing lane from its normal white color to red. There is an RGB LED indicator for each testing lane integrated to alert testing progress to the user. We have also interfaced a 20 × 4 liquid crystal display to the Arduino MEGA. This display is used during testing mode selection and also displays the elapsed time of each mouse at the end of each testing session. The LCD is panel mounted to an opening in the enclosure. The LCD, sensor, and RGB LED wiring diagrams can be seen in Figure 4*C–E*, respectively.

#### The Arduino UNO

The Arduino UNO microcontroller is used to monitor the output voltage of our five infrared range sensors. We use five on-board analog to digital converters for this purpose. If the input voltage of any sensor increases above a threshold, this board will set a digital output line high. This digital output is used as an interrupt by the Arduino MEGA described next. To avoid artificially triggering interrupts by noisy voltage signals, a digital low-pass filter is employed. The Arduino UNO is powered via its V_in_ pin from our 5-V DC power supply mentioned above. Specific pin connections can be observed in Table 3.

**Table 3:** Code variable and pin definitions for microcontrollers

Variable	Definition	Pin
Part 1. Defined pins for main microcontroller		
redLED0	LED 1	34
grnLED0		36
bluLED0		37
redLED1	LED 2	38
grnLED1		40
bluLED1		41
redLED2	LED 3	42
grnLED2		44
bluLED2		45
redLED3	LED 4	46
grnLED3		48
bluLED3		49
redLED4	LED 5	50
grnLED4		52
bluLED4		53
dirPin	Stepper motor	26
pulPin		28
interruptPin0	Receives sensor 1 output	3
interruptPin1	Receives sensor 2 output	18
interruptPin2	Receives sensor 3 output	19
interruptPin3	Receives sensor 4 output	20
interruptPin4	Receives sensor 5 output	21
startButton1Pin	Start pushbutton	24
cycleButtonPin	Cycle pushbutton	22
interruptKillPin	Emergency stop pushbutton	2
radiusPin	Toggle switch (for radius size)	23
Part 2. Defined variables for main microcontroller (Arduino MEGA)		
startTime	Represents time after ramping sequence begins	
endTime0	Time after mouse 1 falls	
endTime1	Time after mouse 2 falls	
endTime2	Time after mouse 3 falls	
endTime3	Time after mouse 4 falls	
endTime4	Time after mouse 5 falls	
elapsedTimeProgress	Used to represent present time throughout the program (used for equations)	
elapsedTime0	Time mouse 1 spend on the rod from ramping program start to when it falls (endTime0-startTime)	
elapsedTime1	Time mouse 2 spend on the rod from ramping program start to when it falls (endTime0-startTime)	
elapsedTime2	Time mouse 3 spend on the rod from ramping program start to when it falls (endTime0-startTime)	
elapsedTime3	Time mouse 4 spend on the rod from ramping program start to when it falls (endTime0-startTime)	
elapseedTime4	Time mouse 5 spend on the rod from ramping program start to when it falls (endTime0-startTime)	
mode	Variable used to represent what ramping program (case) is selected	
delayTMillis	Variable for delay time between each impulse sent to stepper motor (in milliseconds)	
delayTMicros	Variable for delay time between each impulse sent to stepper motor (in microseconds)	
previousState	Variable for cycle button programming	
startButtonState	Start button	
mouse0Fell	Variable used for switch case logic	
mouse1Fell	Variable used for switch case logic	
mouse2Fell	Variable used for switch case logic	
mouse3Fell	Variable used for switch case logic	
mouse4Fell	Variable used for switch case logic	
mouse0Done	Variable used for switch case logic	
mouse1Done	Variable used for switch case logic	
mouse2Done	Variable used for switch case logic	
mouse3Done	Variable used for switch case logic	
mouse4Done	Variable used for switch case logic	
killButtonState	Emergency state button variable	
radiusFactor	Used to adjust equations for rods of different diameter	
radiusSwitchState	For toggle switch logic	
loopCount	For pushbutton logic	
Part 3. Defined pins and variables for secondary microcontroller (Arduino UNO)		
currentVolts0	Calculated volts from sensor 1 based on raw data sent to microcontroller	
currentVolts1	Calculated volts from sensor 2 based on raw data sent to microcontroller	
currentVolts2	Calculated volts from sensor 3 based on raw data sent to microcontroller	
currentVolts3	Calculated volts from sensor 4 based on raw data sent to microcontroller	
currentVolts4	Calculated volts from sensor 5 based on raw data sent to microcontroller	
previousVolts0	Volts from previous data reading	
previousVolts1	Volts from previous data reading	
previousVolts2	Volts from previous data reading	
previousVolts3	Volts from previous data reading	
previousVolts4	Volts from previous data reading	
newVolts0	Volts from sensor 1 after passed through digital lowpass filter	
newVolts1	Volts from sensor 1 after passed through digital lowpass filter	
newVolts2	Volts from sensor 1 after passed through digital lowpass filter	
newVolts3	Volts from sensor 1 after passed through digital lowpass filter	
newVolts4	Volts from sensor 1 after passed through digital lowpass filter	
a	Value for digital low pass filter	
pin 2	Output for sensor 1	
pin 3	Output for sensor 2	
pin 4	Output for sensor 3	
pin 5	Output for sensor 4	
pin 6	Output for sensor 5	

#### The Arduino MEGA

The Arduino MEGA is a more capable microcontroller board than the Arduino UNO with a greater number of input/output pins and memory capacity. The three user-controlled push buttons are connected to this board for initial mode selection and to start each test. The Arduino MEGA also controls the stepper motor movement by sending timed pulses to the stepper motor controller at predetermined intervals. The Arduino MEGA also communicates with the LCD before, during, and after testing. Importantly, the Arduino MEGA measures latency to fall times for each mouse and reports this time to the LCD either at the end of the test or when the user presses the stop button. Timing for fallen mice is measured based on interrupts received from the Arduino UNO as described above. The Arduino MEGA is powered via its V_in_ pin from our 5-V DC power supply mentioned previously. The specific pin connection can be observed in Table 3.

### Code accessibility

The code/software described in the paper is freely available online at https://github.com/MunteanLab/mouseduino. The code is available as Extended Data 1.

10.1523/ENEURO.0123-23.2023.ed1 Extended Data 1 Arduino code and files for 3D printing. Download Extended Data 1, ZIP.

### Program/code

#### Overview

Our custom rotarod device is programmed using the Arduino Integrated Development Environment (IDE) available at no cost at www.arduino.cc. For the purpose of modularity, we have chosen to use two Arduino microcontroller boards for our system as mentioned above. The Arduino MEGA coordinates the stepper motor movement, LCD panel, LEDs, pushbuttons, toggles/switches, and timing data. Conversely, the Arduino UNO functions to measure voltage output from each of five infrared distance sensors. These voltages vary inversely with distance; when there is no mouse in the light path the voltage is low, but a fallen mouse causes a significant voltage increase. This increase is used to trigger an interrupt signal to the Arduino MEGA which then stops the timing for that particular fallen mouse. The key components, structures, language used, and brief instructions on set-up are described below.

#### Set-up

To incorporate the code into the Arduino MEGA, connect a laptop device to the USB input on the microcontroller board using an ordinary printer-type cable. Download the free Arduino IDE software (www.arduino.cc) and code found on our GitHub repository linked above. Open the Arduino IDE and copy the Arduino MEGA code into a new sketch. While the microcontroller is connected, select “upload” (arrow button next to the check button). Repeat this procedure for the Arduino UNO. Change output and input pins as needed based on your wiring strategy.

### Main microcontroller program

#### Overview

The main microcontroller code integrates multiple systems to perform rotarod performance tests. This code manages the following tasks:

1. Provides control of the stepper motor movement which is mechanically connected to the rotarod. (The stepper motor and rotarod may have either constant or variable speed depending on the mode of operation selected by the user.)
2. Enables user mode selection via pushbuttons and an LCD display.
3. Senses, records, and displays the elapsed time or latency after a mouse has fallen.
4. Controls LED colors as a visual cue for the user to recognize a fallen mouse.
5. Integrates a power switch and radius selection toggle switch.

#### Program sections

##### Globals

This section contains initializing variables. First, the link to the liquid crystal library along with its corresponding pins can be observed. RGB LED indicator pins are also defined and labeled with a number from 0, 1, 2, 3, and 4 in correlation with one of the five mouse chambers. Other pins for the stepper motor, interrupt pin, pushbuttons, and other timing and state variables are listed in this section. See the comments for additional details.

##### Interrupt service routines

This section includes function calls which the code will execute in response to receiving an interrupt signal from the Arduino UNO system which, as described earlier, detects fallen mice. Any interrupt will cause the program to break out of its normal sequence and carry out the action of the corresponding interrupt service routine. Using interrupts thus allows for the program to sense a fallen mouse or provide an emergency stop by the kill pushbutton while conducting motor rotation and other operations.

##### void setup ()

This section of the program represents the first sequence performed and will execute once at the beginning of the program. Here, we define the states of each pin as either output, input, or set it up as an interrupt pin. Thus, it acts as a section to set the initializing terms of the program. Aside from setting up pins, it also contains an optional power up light sequence during the device boot-up (which can be deleted if desired) as well as prints an initial start message on the LCD display.

##### void loop ()

The void loop section of the code contains the majority of the program and executes each line of code in order until it reaches its end which causes it to return to the top of the void loop and start over. The beginning of this section is composed of defining initial statements to establish elapsed time and push-button states. This is followed by code that links the cycle button to the LCD display such that different ramping sequences or modes can be cycled through and selected. The main bulk of code is structured in a switch case statement. Within a switch case statement, the program matches up a case with the variable defined in the parenthesis. We defined the variable within the parentheses of switch () as the dynamic variable “mode.” The variable mode is defined by the number of times the cycle pushbutton was pushed, thus allowing the user to control the case (ramping sequence) that would be initiated. Each mode had while loops that had the condition of at least one mouse not fallen or the kill-button not being pressed. See Figure 5 for an overview of the code structure.

### Secondary microcontroller programs

#### Program overview

Our Arduino UNO resembles an electronic comparator; it receives inputs from the five infrared distance sensors. When a mouse falls, this voltage increases above a set calibration level at which the Arduino UNO produces an interrupt to the main Arduino MEGA board. We use the SHARP GP2Y0A02YK0F infrared range sensor (20–150 cm) which generates an analog voltage signal which varies inversely with distance. Thus, in general, closer objects (like fallen mice) will result in a higher voltage (except if too close to the sensor where the object will induce an inappropriate response) which we use to produce the Arduino MEGA interrupts.

#### Program sections

##### Globals

The globals are defined at the top of program. Corresponding definitions for each variable are described in Table 3.

##### void setup ()

Here, we set five pins that correspond to each of the five sensors as an output that will send an interrupt signal to the main microcontroller thus signaling that an object (mouse) has been detected by that sensor.

##### Void loop ()

Within the body of the program, we first convert the analog information from the sensor to voltage. The next section includes a digital lowpass filter used to decrease noise spikes that may have resulted in sporadic and undesired triggers. This is followed by the logic that defines the function of the comparator. It states that the processed voltage value, if higher than 2.0 V (this number can be manipulated based on the sensitivity of your sensors), then send out an output signal (“high”) on the corresponding pin of the microcontroller to alert a mouse has fallen. This output pin is connected to an interrupt pin on the Arduino MEGA that allows for the program to record the time and alert the user when the mouse has fallen.

### Manipulating program equations

#### Setting the stepper motor rotation speeds

The NEMA 23 stepper motor functions based on a sequence of electrical pulses that influence a magnetic field which induces precise single step movements that rotates a shaft which in turn is mechanically coupled to the rotarod apparatus. The rate of rotation of the stepper motor shaft and rotarod is determined by the delay time between electrical pulses. A series of calculations for the delay times based on the desired rotarod rotation speed are performed by the Arduino MEGA program. For more information on determining delay time calculations for ramping and static modes, see “Manipulating Program Equations” attached under “code” in our GitHub repository.

### Animal subjects

All experimental procedures using mice were approved by Augusta University’s Institutional Animal Care and Use Committee (IACUC) in compliance with guidelines set by the National Institutes of Health (NIH). Animals had continuous access to food and water and were maintained under standard housing conditions with a 12/12 h light/dark cycle. Mice used in behavior experiments were from a C57BL/6 background. Testing was performed between two and four months of age. Equal numbers of male and female animals were used in each testing group. The weight for this age range of animals was 21.7 g ± SD of 3.6 g (*n* = 32 mice), which aligns with physiological data for C57BL/6 (RRID:IMSR_JAX:000664). The age of animals in each cohort were (# weeks ± SD): small rod (11.9 ± 2.1), large rod (10.4 ± 1.7), small laddered (10.8 ± 1.5), large laddered (11.5 ± 2.7).

### Behavioral testing

Rotarod performance was examined with an acceleration from 4 to 60 rpm, as similarly described (Muntean et al., 2021, 2022). Testing consisted of three trials per day with at least 5 min between trial intervals. Each trial ended if the mouse fell from the rod, completed two full rod revolutions, or maintained performance for 3 min. The latency to fall was then recorded for each mouse. Animals were consistently tested for 5 consecutive days. Different cohorts were subjected to the four distinct rod designs described above. As similarly described (French et al., 2012; Hirata et al., 2016; Sutton et al., 2019), learning rate was calculated as the mean latency of the first day subtracted from the mean latency of the final day divided by the span of days tested [e.g., (Latency_Final_ – Latency_Initial_)/5 d].

### Data analysis/statistical method

Graphs represent mean ± SEM overlaid with plots of each individual data point as denoted in individual figure legends. Statistical analysis was performed with GraphPad Prism 9 as indicated in appropriate figure legend. Nonparametric Student’s *t* test (Mann–Whitney *U* test) or nonparametric Kruskal–Wallis one-way analysis of variance (Dunn’s multiple comparisons test) was used for experimental comparisons with utilization of symbols to indicate statistical significance as indicated in the appropriate figure legends and Results. A family-wise α threshold and confidence level was set at 0.05 (95% confidence interval).

## Results

**Figure 6.**
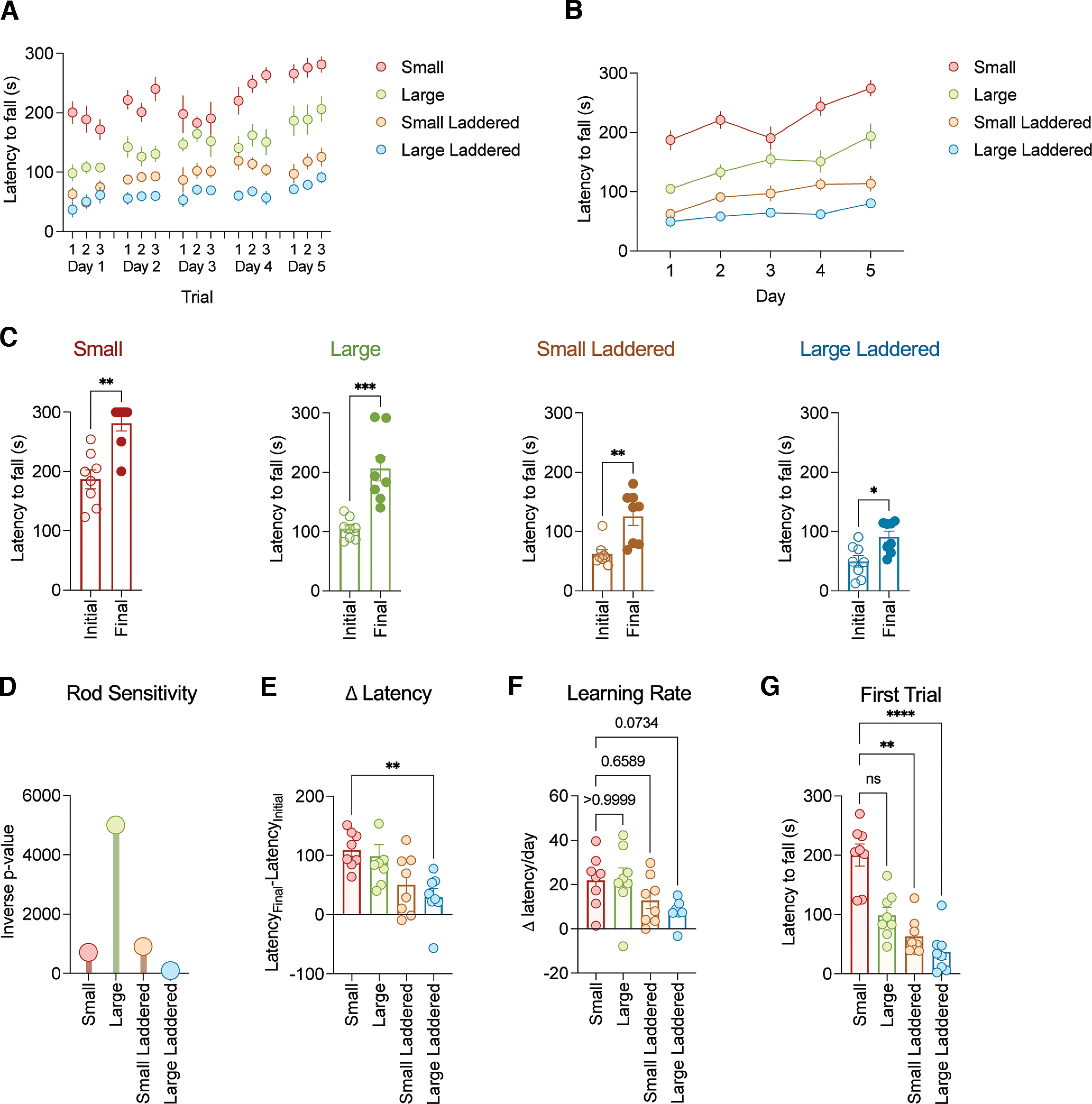
Motor learning test results using different rod sizes and shapes. ***A***, Daily accelerating rotarod performance on each different rod design on intraday intervals. *N* = 8 mice per rod. ***B***, Accelerating rotarod performance on each different rod design over 5 d. *N* = 8 mice per rod. ***C***, Average latency to fall on Initial (day 1) and Final (day 5) days for each rod design. *N* = 8 mice per rod. Nonparametric *t* test (Mann–Whitney test). ***D***, Plot of inverse *p*-value from data in panel ***C***. Small = 1/0.0014, large = 1/0.0002, small laddered = 1/0.0011, large laddered = 1/0.0104. ***E***, Change in latency from Initial (day 1) and Final (day 5) days for each rod design. *N* = 8 mice per rod. Nonparametric Kruskal–Wallis followed by Dunn’s test. ***F***, Accelerating rotarod learning rate for each rod design. *N* = 8 mice per rod. Nonparametric Kruskal–Wallis followed by Dunn’s test. ***G***, Latency to fall from the first trial on day 1 for each rod design. *N* = 8 mice per rod. Nonparametric Kruskal–Wallis followed by Dunn’s test. All data, except panel ***D***, are represented as mean ± SEM; *p < 0.05, **p < 0.01, ***p < 0.001, ****p < 0.0001; ns = not significant (*p* > 0.05).

We tested the utility of our rotarod by examining motor performance on four groups of C57BL/6 mice. Each group contained four males and four females from two to four months of age. The experimental design enabled assignment of one group of mice to each unique rod design: small, large, small laddered, large laddered. We used a relatively standardized accelerating rotarod protocol where mice were placed on a stationary rod that was subsequently programmed to accelerate from 4 to 60 rpms over the span of 5 min (300 s). We then recorded the duration of time that the animal stayed on the rod (latency to fall; Fig. 6*A*). We repeated three intraday trials over 5 consecutive days for each cohort (Fig. 6*B*). Consistent with previous literature, mouse learning improved on each successive day as evidenced by a trending increase in latency to fall. We first compared the average initial (average latency across trials on day 1) and final (average latency across trials on day 5) time on the rotarod (Fig. 6*C*). Mice exhibited the greatest latency on the small rod followed by the large rod with the laddered rods resulting in the lowest latencies (Small: Mann–Whitney *U* test, *U *=* *4, **p = 0.0014, *n* = 8 mice; Large: Mann–Whitney *U* test, *U *=* *0, ***p = 0.0002, *n* = 8 mice; Small laddered: Mann–Whitney *U* test, *U *=* *3, **p = 0.0011, *n* = 8 mice; Large laddered: Mann–Whitney *U* test, *U *=* *8, *p = 0.0104, *n* = 8 mice). Each rod resulted in a statistically significant difference between the initial and final day of testing, which suggests that each rod may successfully examine motor learning. Nonetheless, the large unladdered rod yielded results with the greatest statistical power (Fig. 6*D*). Moreover, the unladdered rods (small and large) exhibited the greatest overall change in latency between initial and final day (Fig. 6*E*; Kruskal–Wallis followed by Dunn’s test, Kruskal–Wallis statistic = 13.4, *p*-value = 0.0037: Small vs Large *p* > 0.9999, *n* = 8 mice each rod; Small vs Small laddered: *p* = 0.0853, *n* = 8 mice each rod; Small vs Large laddered: **p = 0.0057, *n* = 8 mice each rod). Accordingly, the rate of learning was greatest for the unladdered rods with a notably, albeit insignificant, smaller window for the laddered rod designs (Fig. 6*F*; Kruskal–Wallis followed by Dunn’s test, Kruskal–Wallis statistic = 9.028, *p*-value = 0.0289: Small vs Large *p* > 0.9999, *n* = 8 mice each rod; Small vs Small laddered: *p* = 0.6589, *n* = 8 mice each rod; Small vs Large laddered: *p* = 0.0734, *n* = 8 mice each rod). To gauge which rod exhibited the highest sensitivity to initial motor performance, we analyzed latency from the first trial of each mouse (Fig. 6*G*; Kruskal–Wallis followed by Dunn’s test, Kruskal–Wallis statistic = 21.22, *p*-value < 0.0001: Small vs Large *p* = 0.2738, *n* = 8 mice each rod. Small vs Small laddered: **p = 0.0091, *n* = 8 mice each rod. Small vs Large laddered: ****p < 0.0001, *n* = 8 mice each rod). In this parameter we found the small rod delivered the highest sensitivity, which was twofold greater than the large rod and over threefold higher than the laddered designs. Overall, these experiments reveal the large unladdered rod provided the greatest sensitivity for motor learning, the small unladdered rod was ideal to assess initial motor performance, and the laddered designs provided the greatest challenge.

## Discussion

Here, we present the guided assembly of a customizable rotarod device for mouse behavioral testing. Our device was developed using Arduino microcontroller boards that coordinate activity between electrical and physical components. These components work in tandem to permit control over rotational capabilities, accurate latency to fall measurements, and coordinate appropriate prompts for user interaction to perform experiments. This enables all five sensors on the rotarod to operate as a standalone unit not tethered to a computer. Therefore, multiple instruments could be run in parallel providing unprecedented scalability. Our device, both low-cost ($500–$800) and open-sourced, provides a hyperflexible model for mouse performance testing. We created this device to implement unique, custom rod speeds and ramping sequences. Our assembly provides streamlined access to the main microcontroller ports thus allowing computer connections that can be used to quickly update or integrate new programs even within a testing session. These parameters are simply limited by the user’s creative demands as the device can accommodate experiments requiring constant rod speeds, linear and hyperbolic ramping speeds, and sequences of unique backward and forward rotations. Additionally, as labs turn to nonstandard drum sizes to improve the sensitivity of their experiments (Shiotsuki et al., 2010), we expect our device to complement and adapt to these advances as it maintains the ability to detach and replace components of the rod and its subsidiaries. Because of the customizability in both hardware and software, we expect the paradigms of existing motor performance testing to evolve in a more sensitive and specific fashion.

To demonstrate that our open-source approach sufficiently documents motor performance, we compared multiday motor learning between four unique rod designs. Indeed, each rod sufficiently monitored motor learning with unique strengths and weaknesses between designs. Importantly, data collected with our small rod (1.75 inch/4.445 cm diameter) is consistent with prior work on a similar rod design (3 cm in diameter) from commercial vendors (Chen et al., 1995; Costa et al., 2003; Monville et al., 2006; Guyenet et al., 2010; Shiotsuki et al., 2010; Penhune and Steele, 2012; Deacon, 2013; Hirata et al., 2016; Sutton et al., 2019; Muntean et al., 2021, 2022). Moreover, we demonstrated that our larger rod (4 inch/10.16 cm diameter) may increase the sensitivity of the motor learning readout. This is likely because several mice on the small rod reached the 300-s cutoff by the final day of testing. This rarely occurred on the large rod and never on the laddered designs. One reason may be because of the ability of mice to cling to the smaller rod encouraging bouts of passivity, whereas the larger rod requires greater coordination to balance as well as maintaining ongoing momentum (Rozas et al., 1998; Shiotsuki et al., 2010). Therefore, the larger rod may additionally factor traits pertaining to balance, gait, and coordination. Indeed, the laddered rod designs also presented the mice with a greater motor challenge as it required precise reaching to the rungs on the rod. This may be of particular interest for experimental cohorts where standard rod shape are not able to discern differences in motor ability. For instance, animals with dystonic phenotypes often freeze their hind paws in a clasping motion (Guyenet et al., 2010). Clasping would not necessarily mitigate performance on the standard rod, but would present a significant challenge on the laddered design. We are optimistic that the customizable nature of our rotarod will enable such functional investigations that were not previously accessible. In this regard we have also built in additional acceleration ramping rates and paradigms (e.g., forward and backward). We have taken further steps to future-proof our design by including clear acrylic side barriers that enable mounting high-speed cameras for gait or reaching analysis. We are hopeful that these collective improvements to the classic rotarod approach will be of great benefit to the open neuroscience community and simultaneously reduce economic barriers.

## Synthesis

Reviewing Editor: Jonathan Lee, University of Birmingham Decisions are customarily a result of the Reviewing Editor and the peer reviewers coming together and discussing their recommendations until a consensus is reached. When revisions are invited, a fact-based synthesis statement explaining their decision and outlining what is needed to prepare a revision will be listed below. The following reviewer(s) agreed to reveal their identity: Hitoshi Inokawa.

The manuscript was reviewed by one reviewer and the reviewing editor. We agreed that the development of a low cost open source rotarod system is highly valuable and the developed system fits this aspiration. The demonstration of its utility through the presented behavioral data is also a great benefit. To this end, we would strongly recommend that the statistics for these experiments are fully reported in the text. There do appear to be novel findings and the community would greatly benefit from open access to statistical outcomes in order to power planned experiments.

The value of a low-cost open source system is that it might encourage adoption among the wider research community. As such, the introduction and methods would benefit from a little bit more theoretical justification for:

- the unreferenced statement that the current unevolved rotarod is suboptimal for the observation of motor traits across systems

- on what basis the larger diameter drum would be more challenging

Finally, it is not clear whether multiple sets of 5 sensors can be run in parallel from one computer and instance of the running programme. Readers would very likely be interested in scalability when comparing against commercial systems.

The reviewer’s full review is appended below, to which we would encourage a full response in addition to addressing the highlighted points above.

____________________________

In this paper, the authors describe an open source, Arduino-based customizable low-cost rotarod system.

The unique feature is that various rod shapes can be used with a 3D printer, and the speed of rod rotations can be programmed as desired.

The authors were, in fact, able to obtain results with this device that were comparable to those obtained with the previously marketed rotarod tests. In addition, they succeeded in showing differences in the motor learning process depending on the shape of the rotarod.

The low-cost rotarod system with various customizable performance features of this study will undoubtedly contribute to the development of neuroscience related to motor learning.

The paper is very good as it is and I think it is able to publication in eNeuro after minor revisions listed below.

1. Is it possible to provide the data to be formed the parts of the rotarod system by a 3D printer?

2. Why is a nonparametric test used when comparing mean latency to fall between Day 1 and Day 5 and a parametric test when comparing mean latency to fall for 4 different rods? What is the reason for this?

3. Please include statistics in the results of all statistical tests.

4. In the results, since locomotor performance is related to body weight and age in months, please indicate body weight and age in months for each group of mice.

5. Line 103, The static end of the rod (the “right” end as seen on Figure 2B), contains a pulley part (labeled as “top pulley” in our GitHub repository). and Line 107, The dynamic end (the “left” end as seen on Figure 2B). But, in Figure 2A, The cuboid shaft on the front side is labeled as the right piece. Please correct left and right direction.

6. Please insert a space between Voltage and DC.

7. Line 156, displayed on the “liquid crystal display (LCD)”, However, for the first time LCDs are used in Line 63.

8. Table 2, “Linear Potentiometer 10 k”, please insert Ω after 10.

9. Line 89, The abbreviation PLA is shown but not used in the text thereafter.

10. Line 138, DM860A is not shown in table 2

11. All table style is broken. Probably due to the conversion to PDF.
