# Supplementary figures and images for: Customizable Open-Source Rotating Rod (Rotarod) Enables Robust Low-Cost Assessment of Motor Performance in Mice
Source: eNeuro. 2023 Sep 1;10(9):ENEURO.0123-23.2023. doi: 10.1523/ENEURO.0123-23.2023 (PMC10484359; doi:10.1523/ENEURO.0123-23.2023)

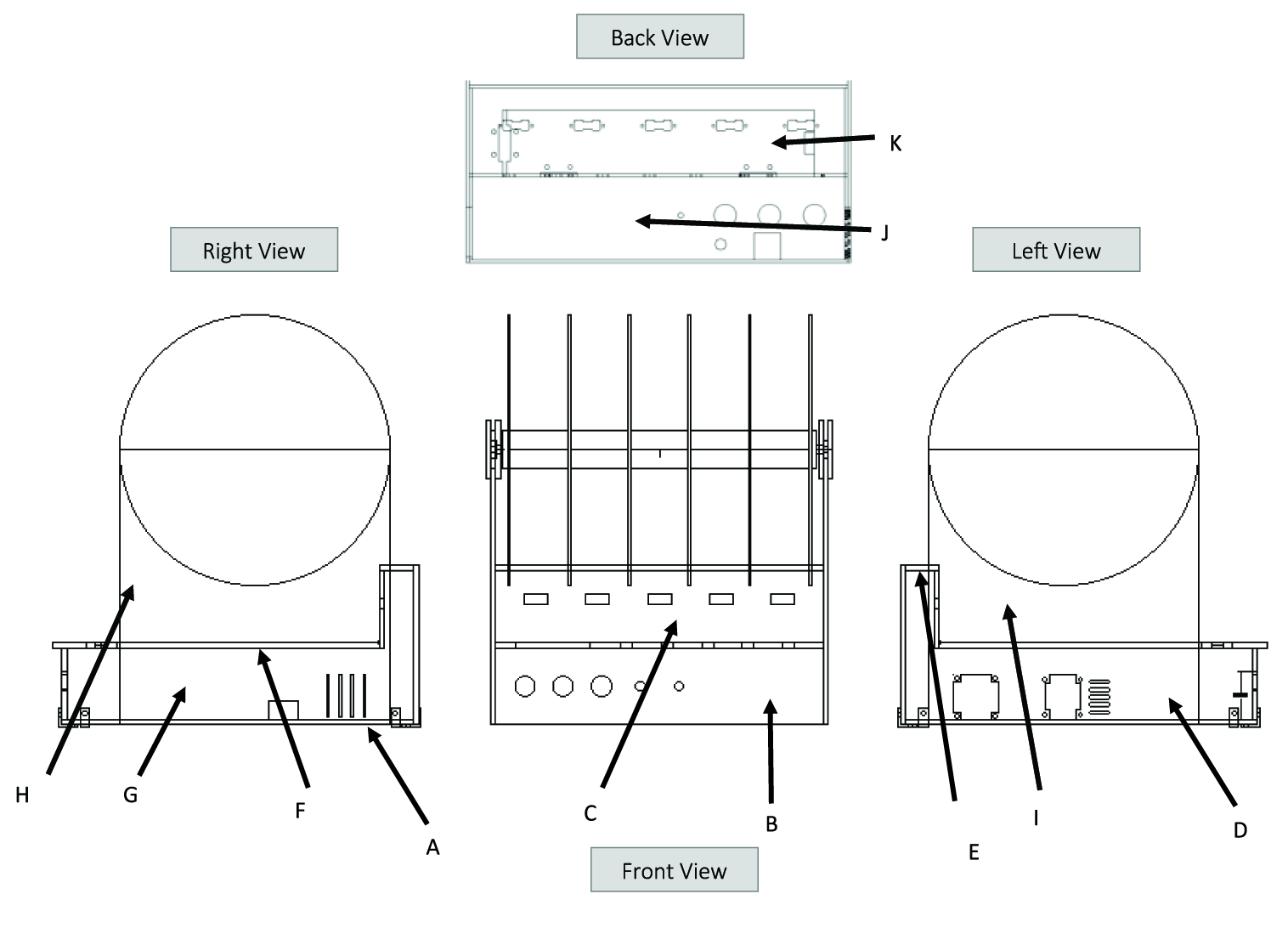

Supplement: Extended Data Figure 1-1 — Base design assembly. Schematic overview of the base with views from the right, left, and back. Download Figure 1-1, TIF file. [file enu-eN-OTM-0123-23-s03.tif]

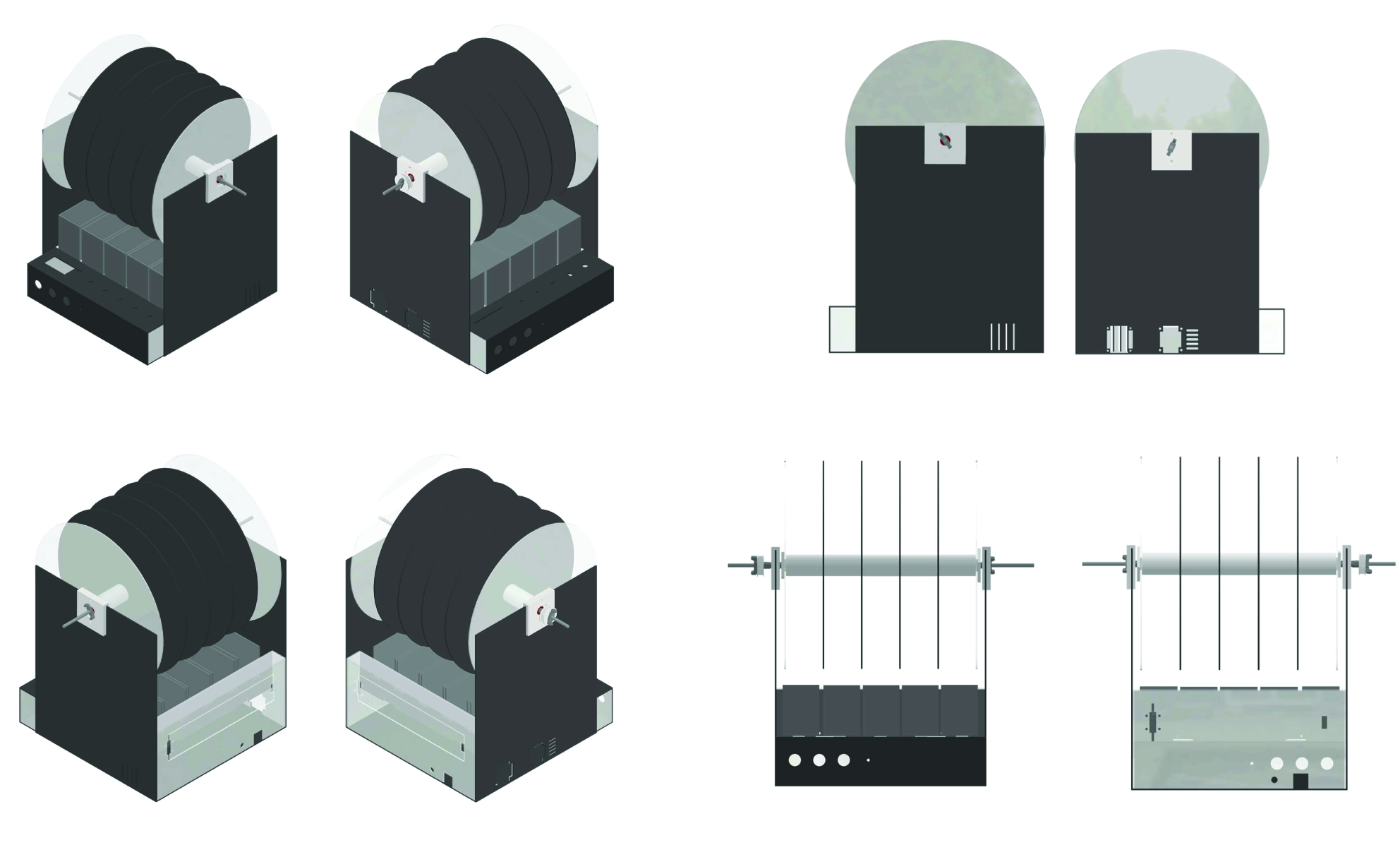

Supplement: Extended Data Figure 1-2 — Expanded schematic views of base rotarod. Download Figure 1-2, TIF file. [file enu-eN-OTM-0123-23-s04.tif]

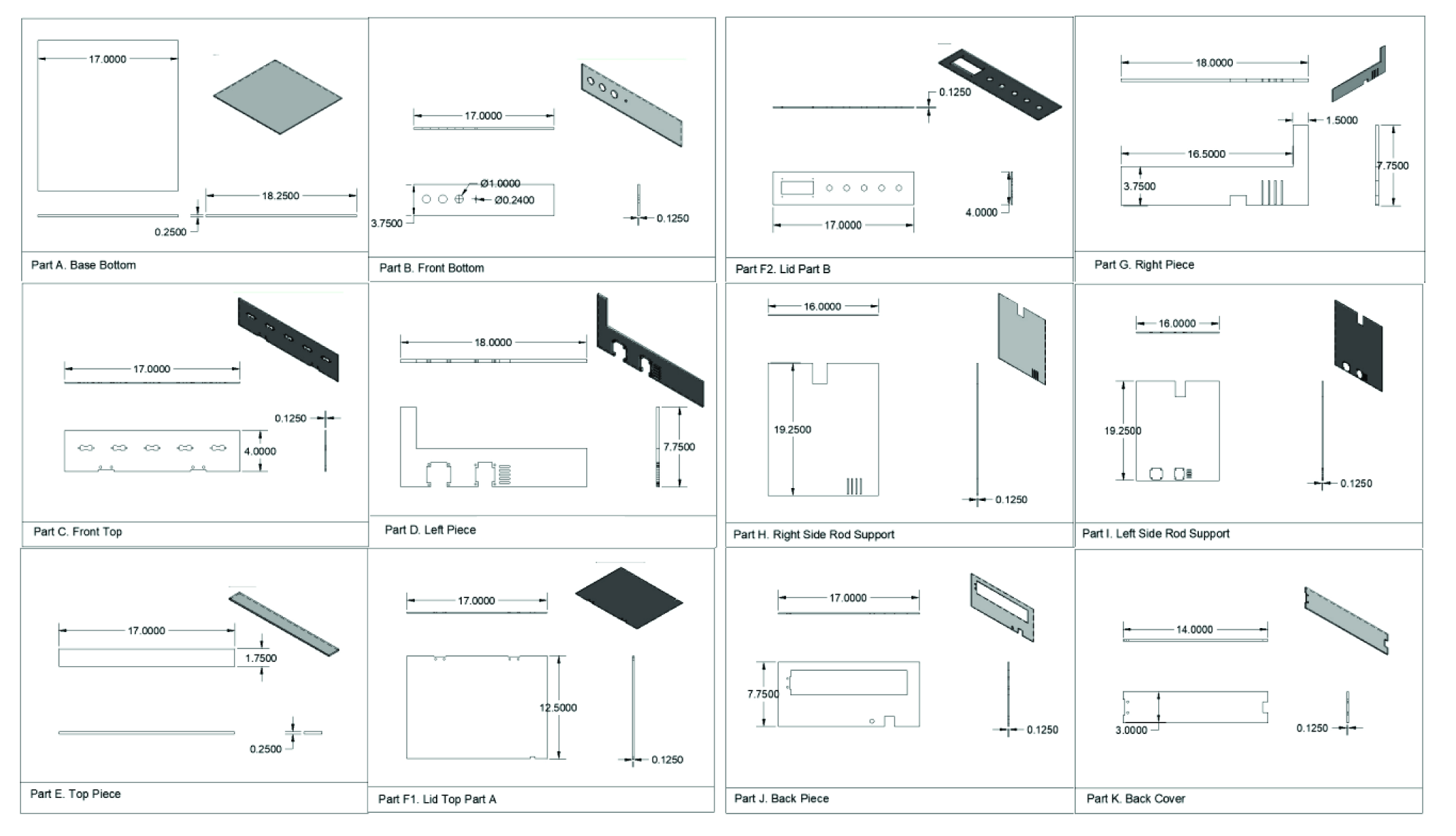

Supplement: Extended Data Figure 1-3 — Labeled components on the base. Download Figure 1-3, TIF file. [file enu-eN-OTM-0123-23-s05.tif]

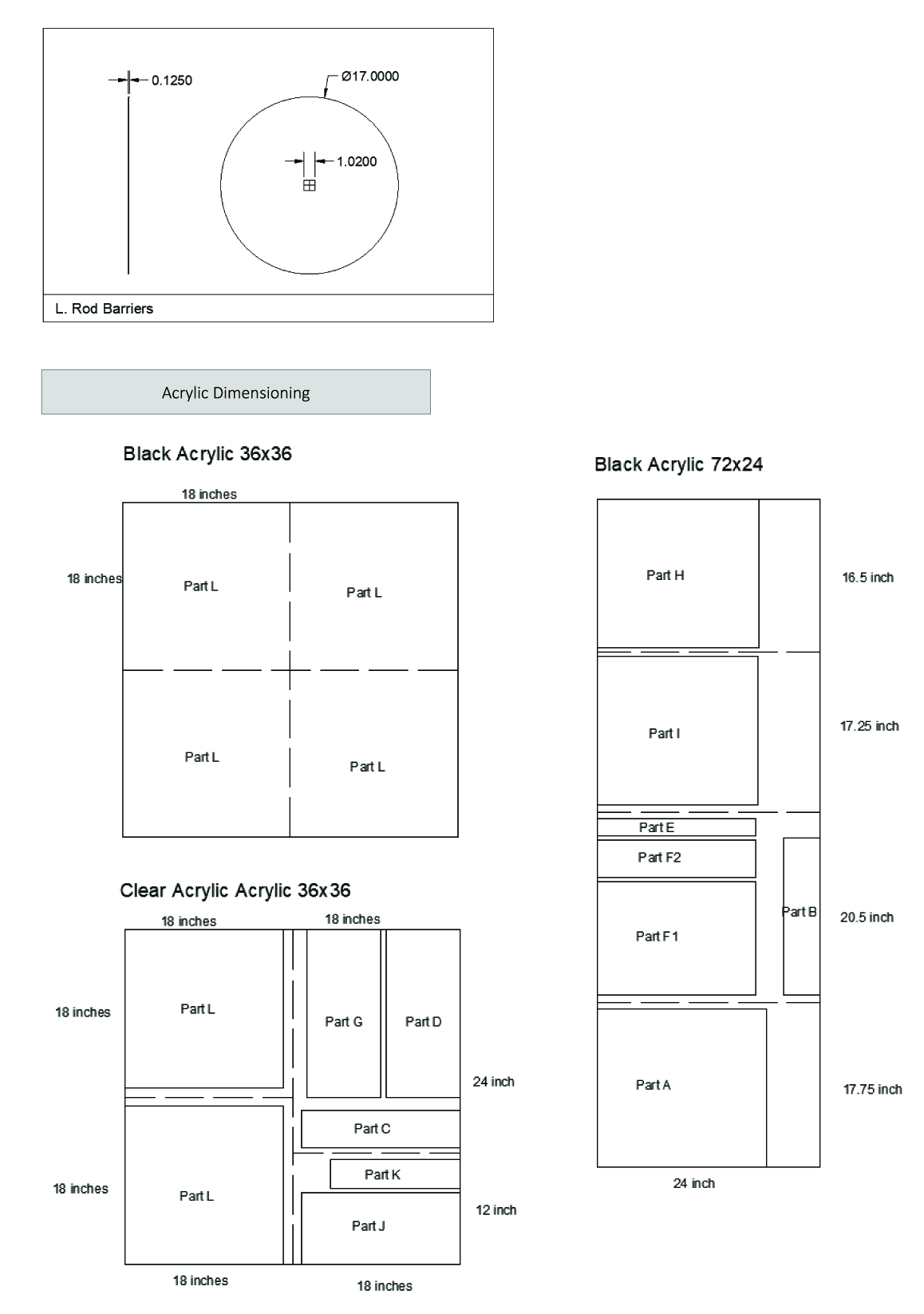

Supplement: Extended Data Figure 1-4 — Rod barriers. The barriers on the rod are shown in part L. The design requires six rods with recommendation for two clear and four opaque barriers. Acrylic dimensioning is also shown. Acrylic sheets were cut down to fit inside our laser cutter. Download Figure 1-4, TIF file. [file enu-eN-OTM-0123-23-s06.tif]

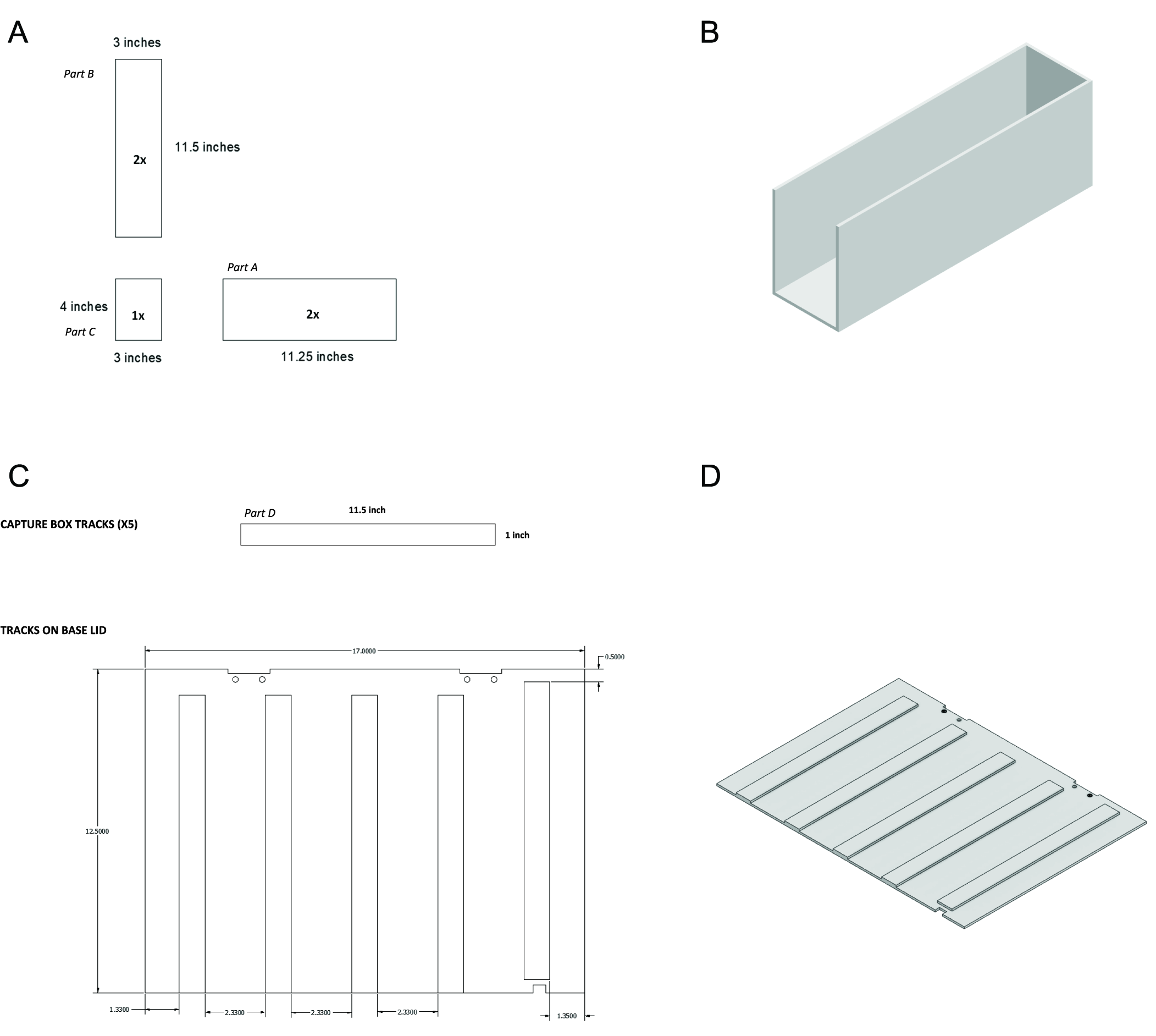

Supplement: Extended Data Figure 1-5 — Mouse capture box design. A, Mouse capture boxes shown with dimensions and were cut from 1/8-inch acrylic sheets. A total of five are required. B, Final view of an assembled capture box. C, Mouse capture box tracks shown with dimensions and were cut from 1/8-inch acrylic sheets. A total of five is required. D, Final view of tracks for the capture boxes. Download Figure 1-5, TIF file. [file enu-eN-OTM-0123-23-s07.tif]

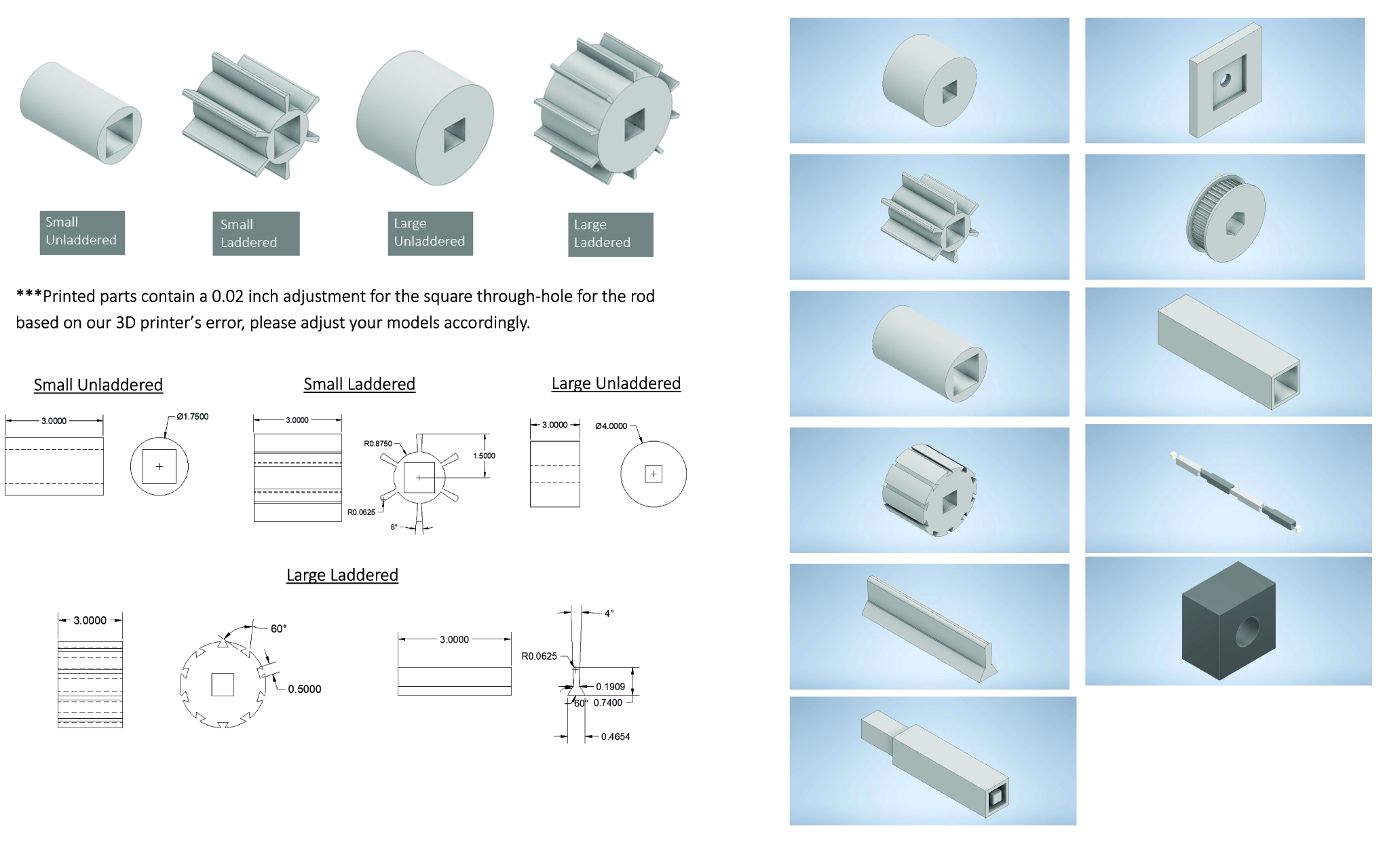

Supplement: Extended Data Figure 2-1 — Rod attachment designs. Designs for the four unique rod models developed. Note that the large-laddered rod was printed in one large piece and ten notched pieces. The notches piece dimensions were synthesized by cutting the notched pieces from a nonseparated laddered rod drawing. Download Figure 2-1, TIF file. [file enu-eN-OTM-0123-23-s08.tif]

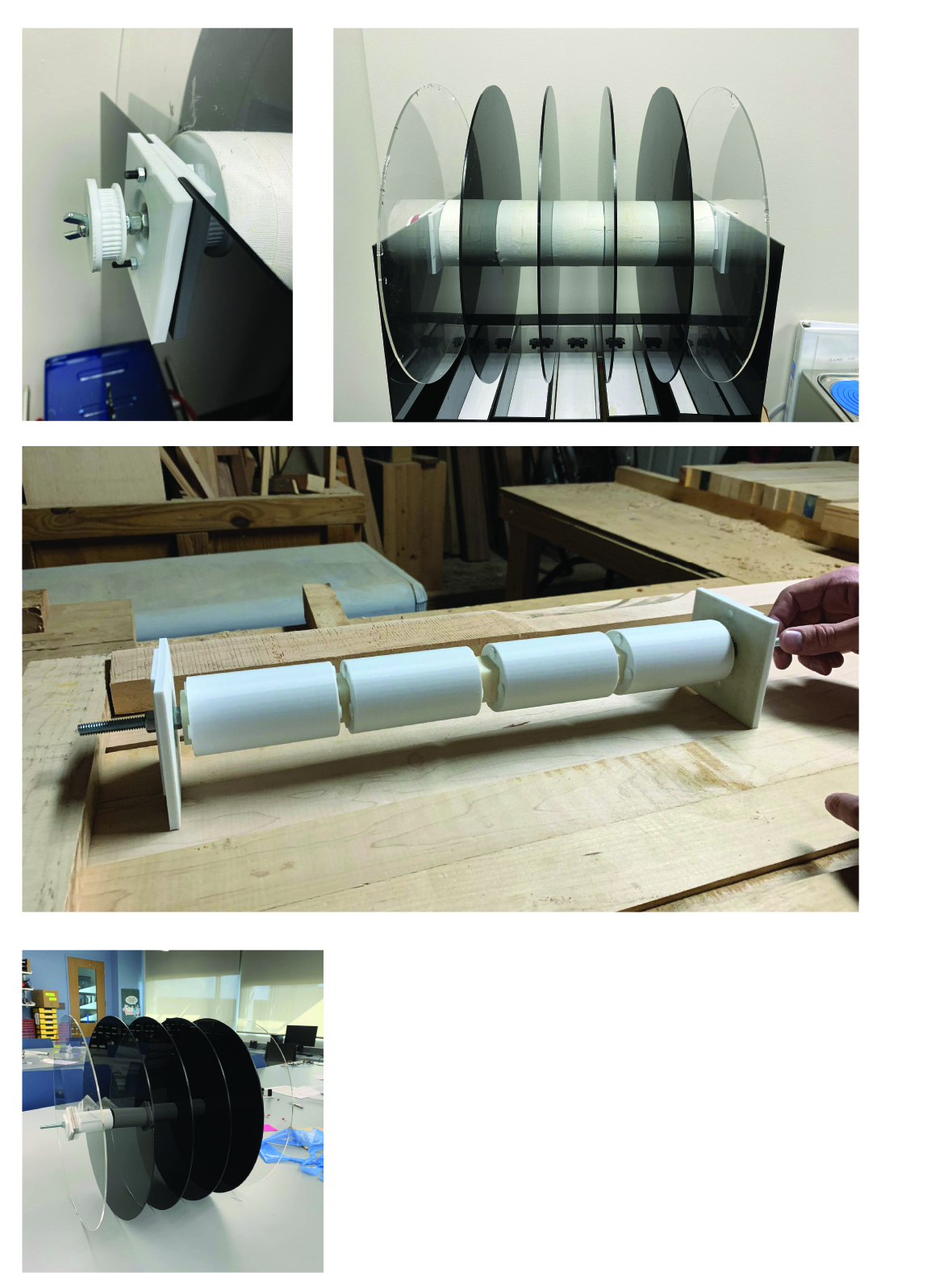

Supplement: Extended Data Figure 2-2 — Actual images of rod assembly. Download Figure 2-2, TIF file. [file enu-eN-OTM-0123-23-s09.tif]

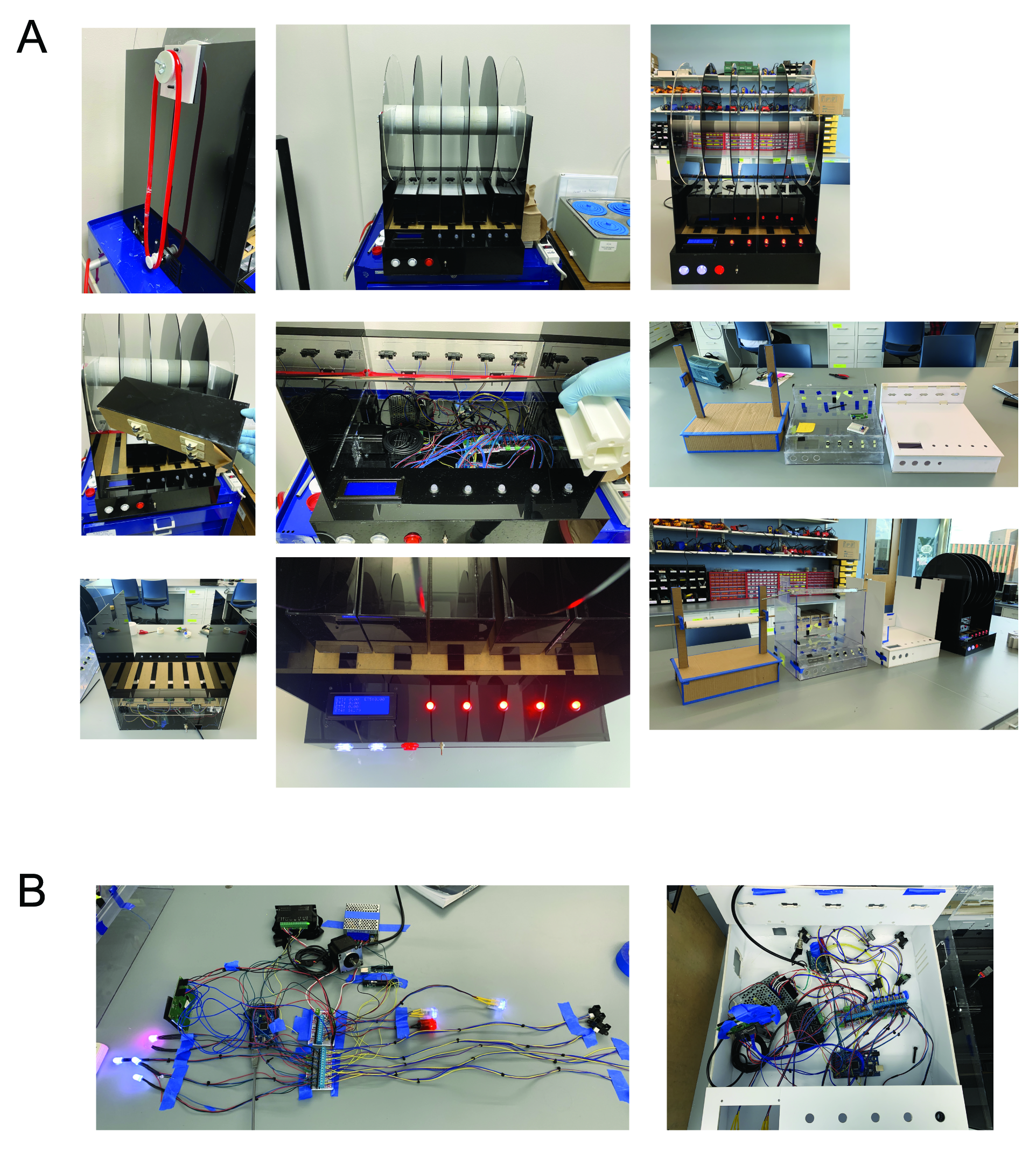

Supplement: Extended Data Figure 4-1 — Actual images of rotarod assembly. A, Images of base assembly. B, Images of electronics assembly. Download Figure 4-1, TIF file. [file enu-eN-OTM-0123-23-s10.tif]
